# MDMA-assisted therapy and current treatment options for chronic, treatment-resistant, moderate or higher severity post-traumatic stress disorder: Systematic literature review

**DOI:** 10.1371/journal.pone.0327778

**Published:** 2025-07-16

**Authors:** Filip Stanicic, Vladimir Zah, Dimitrije Grbic, Djurdja Vukicevic, Debra de Angelo

**Affiliations:** 1 ZRx Outcomes Research Inc., Cawthra Rd, Mississauga, Canada; 2 Lykos Therapeutics, Stevens Creek Blvd, San Jose, California, United States of America; University of Milano–Bicocca: Universita degli Studi di Milano-Bicocca, ITALY

## Abstract

**Background:**

3,4-methylenedioxymethamphetamine-assisted therapy (MDMA-AT) is currently being evaluated for treatment of patients with moderate or higher severity post-traumatic stress disorder (PTSD).

**Objective:**

To provide a comprehensive summary of investigational MDMA-AT and current treatments for PTSD.

**Methods:**

A search was conducted in PubMed and Embase (December 20, 2023). Populations included adults with chronic, treatment-resistant, moderate or higher severity PTSD. Interventions were MDMA-AT and comparators based on PTSD treatment guidelines. The primary outcome of interest was the Clinician-Administered PTSD Scale (CAPS) score. Other outcomes observed were Beck Depression Inventory (BDI), and loss of diagnosis (LOD). Studies observing chronic, moderate or higher severity treatment-resistant PTSD in adults were included. Only randomized controlled trials published in English were considered. The NICE quality appraisal checklist was used to assess risk of bias in included studies. We provided qualitative synthesis of evidence presented in extraction tables.

**Results:**

Overall, 77 studies were included. Phase II/III trials consistently reported significantly greater CAPS improvement with MDMA-AT vs. placebo with therapy (PT) after two or three interventional sessions. Durability was observed in a long-term follow-up trial (mean duration, 45.4 months) with a 0.9-point CAPS decrease from post-treatment.

FDA-approved and off-label medications used for PTSD treatment did not yield a consistently greater CAPS decrease vs. control arms across trials. Significant CAPS improvement was consistently observed in venlafaxine ER, olanzapine, propranolol (with traumatic memory reactivation), nefazodone, and nabilone placebo-controlled trials. Most psychotherapy trials lacked between-group statistical assessments. Significant CAPS decrease compared to the waitlist was reported for cognitive therapy (CT), cognitive behavioral therapy (CBT), cognitive processing therapy (CPT), prolonged exposure (PE), and group cognitive exposure therapy. CAPS improvement was persistent for CPT and PE in long-term follow up (mean duration 6.2 years).

MDMA-AT demonstrated significant improvement in BDI-II score compared to PT (19.7-point vs. 10.8-point decrease, respectively; p = 0.003). The percentage of participants with LOD after two or three active-dose MDMA-AT sessions ranged from 41.7–83.3%.

**Conclusion:**

This systematic review suggests current treatments for PTSD are associated with heterogeneous evidence and the majority do not demonstrate sustained effects. Results from MDMA-AT showed consistent improvements in CAPS, BDI and LOD.

## Introduction

Post-traumatic stress disorder (PTSD) is a psychiatric syndrome caused by direct or indirect exposure to real or threatened traumatic events [1,2]. The most common causes of PTSD are combat-, sexual-, and witness of death or severe injury-related events that lead to flashbacks, avoidance, negative changes in cognition and mood, and alterations in arousal and reactivity. Symptoms can include fear, helplessness, anxiety, and sleep disturbance [3]. Patients with pre-existing factors such as female sex, mental illness diagnosis, and low socioeconomic status are more likely to develop PTSD after experiencing a traumatic event [1,4]. Chronic PTSD develops in individuals who fail to recover from a previously experienced traumatic event and is often combined with depression, substance use disorder, or anxiety [2,3].

According to the Department of Veterans Affairs (VA), approximately 13 million adults are diagnosed with PTSD in the US [5]. The lifetime prevalence of PTSD is about 6.0% and is two times more likely to occur in adult women than men [6]. Veterans and active-duty military personnel are considered high-risk PTSD populations mostly due to combat- and sexual-related traumas [7]. PTSD is also one of the mental health conditions with the highest healthcare costs in the US. About 1.2 million adults are receiving disability benefits due to PTSD diagnosis. The estimated annual economic burden of PTSD for 2018 was $232.2 billion, with a $19,630 cost per individual. The main cost drivers were direct healthcare costs, unemployment among civilians, and disability in military personnel [8].

Treatment of PTSD consists of two main components – psychotherapy and pharmacotherapy [2]. The American Psychological Association (APA) clinical practice guideline for the treatment of PTSD strongly recommends the use of cognitive behavioral therapy (CBT), cognitive processing therapy (CPT), cognitive therapy (CT), and prolonged exposure (PE) for adult patients [9]. Pharmacotherapy aims to reduce symptom severity, mainly depression and anxiety [2]. It includes several different therapeutic classes that are mainly used for the treatment of other psychiatric disorders. Medications may be used independently or in combination with psychotherapy sessions. Some recommended monotherapies for adult patients with PTSD are sertraline, paroxetine, and several off-label medications [9].

Although there are available treatments for PTSD, limitations have been associated with their use [10–13]. High treatment failure rates are observed among first-line psychotherapies and medications. Many patients who have responded to these treatments still retain a PTSD diagnosis [10–12]. For both psychotherapy and pharmacotherapy treatments, patients may also require long-term exposure to maintain effectiveness which often results in adverse events (AEs), treatment dropout, and symptom severity outbreaks [10–13]. The large PTSD burden from both payer and societal perspectives points out the need for new treatment options in patients who do not tolerate or respond to first-line therapies.

3,4-methylenedioxymethamphetamine-assisted therapy (MDMA-AT) is a novel PTSD treatment that synergistically combines the therapeutic effects of MDMA with manualized psychotherapeutic approaches [14]. Published evidence from phase III randomized controlled trials (RCTs) suggest that MDMA-AT has potential to address unmet need surrounding PTSD treatment in clinical practice [15,16]. Therefore, MDMA-AT may lead to a significant reduction of the overall disease burden of PTSD to payers and society.

The main objective of this systematic literature review (SLR) is to provide a comprehensive overview of efficacy and safety reported in RCTs of currently available therapies (psychotherapies and medications) and MDMA-AT, an investigational treatment option, in patients with chronic, treatment-resistant, moderate or higher severity PTSD.

## Methodology

### Data sources and selection criteria

Medical Literature Analysis and Retrieval System Online (MEDLINE®) was the key literature database used, assessed via PubMed and Embase. As the main outcomes were related to the efficacy and safety of various treatments for PTSD management, no time limitations were applied to collect all relevant publications within the existing literature. In addition, a hand search was performed across publicly available domains and reference lists to ensure all relevant studies were included. Only RCTs with intervention and at least one comparator arm including PTSD treatment options of interest, placebo, waitlist, or treatment as usual, were considered. Detailed selection criteria are shown in Table 1.

**Table 1:** Study selection criteria.

Inclusion criteria	Exclusion criteria
Chronic PTSD Moderate or higher severity PTSD Treatment-resistant PTSD Adult patients only Study arms with relevant comparators* Studies with outcomes of interest* Publications written in English	Systematic and narrative reviews Direct and indirect treatment comparisons Non-randomized, single-arm, and observational studies Cross-sectional, case-report, and case-series studies Surveys, physician interviews, and questionnaires Preclinical, *in vitro*, animal, molecular, and genetic studies Guidelines, books, editorials, comments, replies, and letters

* **Note**: Relevant comparators and outcomes of interest are defined in the Search strategy section

### Search strategy

Search queries (S1 Table) were constructed to capture the efficacy and safety evidence of MDMA-AT and other available PTSD treatment options. The search was based on the research question defined by Population, Intervention, Comparators, Outcomes, and Study design (PICOS) criteria (Table 2). The population of interest was adult patients with chronic, treatment-resistant, moderate or higher severity PTSD. Moderate or higher severity was estimated based on the baseline values of PTSD scoring systems (i.e., CAPS-IV ≥ 40 points, CAPS-5 ≥ 23 points) [17]. Disease severity, treatment resistance, chronicity, and age ≥ 18 years criteria were not included in the queries due to the insufficient sensitivity of database search algorithms. Patients were considered treatment-resistant if the study clearly stated this fact or if the patients had another PTSD treatment before enrollment but lacked treatment response (i.e., patients still had severe PTSD despite receiving treatment). Therefore, these criteria were applied during the title, abstract, and full-text screenings. If not explicitly mentioned in the eligibility criteria section, these characteristics were sought in patient characteristics tables and descriptives. Mean PTSD severity CAPS-IV ≥ 40 points, CAPS-5 ≥ 23 points scores were considered eligible. MDMA-AT was the primary intervention, while psychotherapy and medication comparators were selected based on PTSD treatment guidelines [18–21]. Studies were included only if at least two comparators are relevant. In case of additional comparators in the study, article was included, but the data were not extracted for irrelevant intervention. Outcomes of interest were chosen from the reported measures in clinical trial publications of the main intervention. Studies reporting on the outcomes of interest per each treatment arm were included, regardless of the type of the measure, time points, type of analysis, and result reporting (data were also extracted from provided tables and figures). Studies reporting only effect measures, with no data per each treatment, were not included.

**Table 2:** PICOS criteria for the SLR.

PICOS	Description
**Population**	1. Adult patients with chronic, treatment-resistant, moderate or higher severity PTSD
**Intervention**	1. MDMA-assisted therapy
**Comparators**	1. Psychotherapies (CBT, CPT, PE, trauma-focused, and EMDR)
	2. FDA-approved medications for PTSD (sertraline and paroxetine)
	3. Off-label medications for PTSD (fluoxetine, venlafaxine, escitalopram, nefazodone, imipramine, amitriptyline, mirtazapine, phenelzine, brofaromine, risperidone, quetiapine, olanzapine, topiramate, lamotrigine, tiagabine, ganaxolone, divalproex, ketamine, prazosin, propranolol, mifepristone, D-cycloserine, cyclobenzaprine, cannabidiol, dronabinol, bupropion, buspirone, citalopram, desvenlafaxine, eszopiclone, pregabalin, rivastigmine, and duloxetine)
	4. Psychotherapy and PTSD medication combinations 5. Placebo, waitlist, or treatment as usual controls
**Outcomes**	1. Clinical scores (CAPS, BDI, DES, SDS, C-SSRS)
	2. Disease course (relapse, remission, progression, regression) 3. Treatment patterns (dosing, adherence, compliance, persistence, treatment gaps, therapy augmentation, concomitant medications, treatment duration, treatment transition, treatment failure, dropout)
	4. Adverse events and toxicities
	5. Mortality, survival, and suicide rates
**Study Design**	1. Randomized controlled trials
	2. Secondary analyses of randomized controlled trials data

Abbreviations: EMDR – Eye Movement Desensitization and Reprocessing; MDMA – 3,4-methylenedioxymethamphetamine; FDA – Food and Drug Administration; CAPS – Clinician-Administered PTSD Scale; BDI – Beck Depression Inventory; DES – Dissociative Experience Scale; SDS – Sheehan Disability Scale; C-SSRS – Columbia Suicide Severity Rating Scale

### Literature review and data synthesis

The review was conducted in accordance with Preferred Reporting Items for Systematic Reviews and Meta-Analysis (PRISMA) guidelines [22]. Two independent reviewers performed the database search, abstract and title review, full-text screening, and data extraction. A third reviewer resolved any disagreements. Predefined extraction tables were used for data collection and evidence summary. For the ease of navigation and comparison, study intervention characteristics and outcomes were grouped and tabulated according to the relevant interventions (MDMA, psychotherapy, FDA-approved medication, off-label medication). Data were extracted in the original form, as reported in included studies, without summary synthesis or data conversion. A few studies reported outcomes of interest only as figures. In this case, a plot reading software was used to retrieve numerical score points (i.e., automeris.io). Outcomes of the studies that did not report summary statistics were not presented in the efficacy summary, as unbiased synthesis of the evidence was not possible. The results were visually displayed as summary tables. Qualitative evidence synthesis was also provided in the narrative form. Due to the extensive heterogeneity of interventions and reported outcomes, quantitative evidence synthesis was not performed.

The quality appraisal checklist for quantitative studies proposed by the National Institute for Health and Care Excellence (NICE) was used by two independent reviewers to assess and grade each study included in the extraction process (S2 Table). A consistent study evaluation during the double assessment performed on a random 10% sample (8 studies) ensured high accuracy and agreement between the reviewers.

## Results

**Fig 1:**
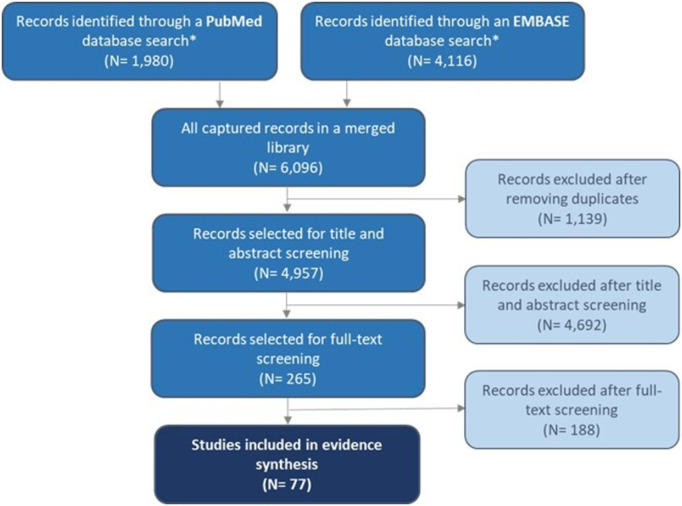
PRISMA flow chart.

A literature search with predefined queries yielded an overall 6,096 hits. After duplicate removal, title and abstract screening was performed on 4,957 studies. There were 4,692 records excluded during this phase, leaving 265 studies for full-text screening. After the evaluation, a final sample of 77 studies was included in the quality assessment, data extraction, and evidence synthesis (study characteristics are provided in S3 Table). PRISMA flow diagram of the literature review process is presented in Fig 1.

### Clinical scores

#### MDMA-AT.

The SLR captured a total of eight MDMA-AT publications with different study designs. There were four phase II trials [23–26], one follow-up study of the phase II trials [27], and two phase III studies with a separately published subgroup analysis [15,16,28].

All MDMA-AT studies were studied across civilian, active duty, first responders, and veteran populations with various types of index trauma, and had similar patient inclusion/exclusion criteria and treatment regimens. Most trials included patients with unspecified trauma types, while Mithoefer et al. studies enrolled only participants with military service-related PTSD (e.g., war-related, crime-related) [23,24,27]. PTSD mean duration was between 14–20 years in all studies except for Mithoefer et al. [24] and Ot’alora et al. [25], with around 7 and 29 years duration, respectively. Length of follow up across MDMA-AT trials ranged from 12 weeks to 18 weeks, except for the long-term follow-up study of phase 2 completers (up to 74 months) [27].

All MDMA-AT studies evaluated PTSD severity using the Clinician-Administered PTSD Scale (CAPS). The summary of CAPS score changes after treatment with MDMA-AT is shown in Table 3.

**Table 3:** Summary of post-treatment CAPS score changes from MDMA-AT publications.

Author	Study Phase	Time Endpoint	Intervention	CAPS Type	CAPS Change	P-value
Mitchell [15]	Phase 3	18 weeks	**120-180 mg MDMA-AT** ^*^	CAPS-5^1^	−24.4 points	**vs. baseline** p < 0.0001 **between-group** p < 0.0001
			Placebo + AT		−13.9 points	
Mitchell [16]	Phase 3	18 weeks	**120-180 mg MDMA-AT** ^†^	CAPS-5^1^	−23.7 points	**between-group** p < 0.001
			Placebo + AT		−14.8 points	
Mithoefer [23]	Phase 2	16 weeks	**125-187.5 mg MDMA-AT**	CAPS-IV	−53.7 points	**vs. baseline** p < 0.0005 **between-group** p = 0.015
			Placebo + AT		−20.5 points	
Mithoefer [27]	Phase 2	17.0-74.0 months	**125-187.5 mg MDMA-AT**	CAPS-IV	−0.9 points	**vs. posttreatment** p = 0.910
Mithoefer [24]	Phase 2	16 weeks	**125-187.5 mg MDMA-AT**	CAPS-IV	−44.3 points	125 mg **vs. 30 mg** p = 0.004 **75 mg vs. 30 mg** P = 0.0005 **125mg vs 75 mg** p = 0.185
			75-112.5 mg MDMA**-**AT		−58.3 points	
			30-45 mg MDMA**-**AT		−11.4 points	
Oehen [26]	Phase 2	12-14 weeks	**125-187.5 mg MDMA-AT**	CAPS-IV	−15.6 points	**Full dose vs. baseline** p = 0.002 **Active placebo vs. baseline** p = 0.475
			25-37.5 mg MDMA**-**AT		3.1 points	
Ot’alora [25]	Phase 2	12 weeks	**125-187.5 mg MDMA-AT**	CAPS-IV^1^	−37.0 points	**125-187.5 mg vs. 40 mg** p = 0.010 **100-150 mg vs 40 mg** p = 0.10
			**100-150 mg** MDMA**-**AT		−24.4 points	
			40-60 mg MDMA**-**AT		−4.0 points	

* The 120–180 mg was a split dose of 80 + 40 mg for the first session and 120 + 60 mg for the second and third experimental sessions. Six participants chose either not to take the supplemental dose (n = 3, 1 MDMA) or not to escalate to the 120 mg dose (n = 3, 2 MDMA) in a total of six experimental sessions (2.3% of the total sessions across the study) [15].

† *Three participants did not undergo dose escalation in sessions 2 and 3* [16].

***Abbreviation:*** *AT – Assisted Therapy; MDMA – 3,4-methylenedioxymethamphetamine; CAPS – Clinician-Administered PTSD Scale*

^1^ *Per-protocol set (completers analysis)*

RCTs consistently reported CAPS improvement after two or three active-dose MDMA-AT sessions in civilian, active duty, and veteran populations with various types of index trauma. CAPS score decreases in phase II trials were 37.0–53.7 points in the 125 mg MDMA arms. These changes were statistically significant compared to baseline and when compared to placebo and low-dose groups [23–25]. The exception was a small sample (12 participants), underpowered trial by Oehen et al. that showed a 15.6-point change in CAPS from baseline (p = 0.002); however, between-group statistics were not reported [26]. Placebo-controlled phase III trials demonstrated mean CAPS score decreases between 23.7–24.7 points, with a significant improvement compared to placebo [15,16]. Additionally, the durability of the CAPS score improvement was observed in a long-term follow-up trial (mean duration 45.4 months) with a 0.9-point CAPS decrease from post-treatment [27].

Several studies observed short- and long-term improvements in depression symptoms by treatment with 120–180 mg or 125–187.5 mg dose MDMA-AT compared to placebo or active controls [15,24,25]. A phase III trial of patients with severe PTSD showed a significantly higher decrease in Beck Depression Inventory II (BDI-II) score from baseline to 18 weeks after three MDMA-AT sessions compared to placebo with therapy among completers (mean 19.7-point decrease from 30.5 and 10.8-point decrease from 34.9, respectively; p = 0.003) [15]. Two phase II trials reported a statistically significant change in BDI-II score from baseline to 12-month follow up in patients who received 125–187.5 mg dose MDMA-AT sessions (both p < 0.0001) [24,25].

Two phase III trials by Mitchell et al. demonstrated significant reduction in functional impairment measured by the Sheehan Disability Scale (SDS) [15,16]. In the first trial which assessed SDS in patients with severe PTSD, the mean change in SDS from baseline to ~18 weeks after baseline was −3.1 and −2.0 among completers for the MDMA-AT and placebo with therapy arms respectively (p = 0.0166). The second phase III trial in patients with moderate or higher PTSD showed similar results, with −3.3 and −2.1 SDS score reductions from baseline to 18 weeks after baseline for the MDMA-AT and placebo with therapy arms, respectively (p = 0.030) [16].

There was a slight variance in the results of MDMA-AT treatment on the Dissociative Experiences Scale II (DES-II) score among the captured publications. Mithoefer et al. [24] reported a significant change in mean DES-II score from baseline after two 75 mg or two 125 mg MDMA-AT sessions (−8.6 and −8.8 change from baseline; p = 0.020 and p = 0.010, respectively) compared to 30 mg MDMA-AT (1.8 change from baseline). Following three MDMA-AT sessions, the mean DES-II score was 5.4 points lower at the 12-month follow up than at baseline (p = 0.046) [24]. Conversely, Ot’alora et al. [25] observed insignificant between-group differences in DES-II score changes from baseline after two 40 mg, 100 mg, or 125 mg MDMA-AT sessions (−0.2, −13.3, and −5.9 points, respectively; p = 0.150). However, a significant change in DES-II from baseline to the 12-month follow-up after three 100−125 mg MDMA-AT sessions was demonstrated (−16.7 points, p < 0.001) [25], with a score change much higher than in Mithoefer et al. [24].

#### Psychotherapies.

Overall, there were 20 studies included in the SLR that evaluated the efficacy (measuring effects via the relevant clinical scores) of psychotherapies in the treatment of chronic, moderate or higher severity PTSD. Per psychotherapy type, nine RCTs [29–37] investigated different modalities of CBT, eight captured clinical trials of PE [36–43], seven observed CPT-treated patients [40–46], three evaluated EMDR [47–49] and two were waitlist-controlled trials to assess outcomes in patients receiving CT [50,51]. Across psychotherapy studies that evaluated efficacy via CAPS scores, the follow-up length ranged from 5 weeks to 6 months, except for one long-term observation with an average duration of 6.15 years [43]. A brief overview of psychotherapy efficacy in PTSD treatment, defined as a change in CAPS score, is presented in Table 4.

**Table 4:** Summary of post-treatment CAPS score changes from clinical trials with psychotherapies.

Author	Study Phase	Time Endpoint	Intervention	CAPS Type	CAPS Change	P-value
Bryant [30]	NR	6 months	**CBT-brief exposure**	CAPS-IV	−39.7 points	NR
			**CBT-prolonged exposure**		−38.4 points	
			Waitlist		−11.9 points	
McDonagh [31]	NR	14 weeks	**CBT**	CAPS-IV	−16.8 points	NR
			Waitlist		−6.5 points	
Fecteau [33]	NR	5 weeks	**CBT**	CAPS-2	−33.4 points	**between-group** p < 0.010
			Waitlist		−2.7 points	
Monson [32]	NR	12-15 weeks	**CBCT**	CAPS-IV	−35.42 points	NR
			Waitlist		−12.20 points	
Beck [34]	NR	14 weeks	**Group CBT**	CAPS-IV	−28.4 points	NR
			Minimum contact group		−8.4 points	
Castillo [38]	NR	16 weeks	**Group-Delivered Cognitive/** **Exposure Therapy**	CAPS-IV	−24.4 points	**between-group** p < 0.001
			Waitlist		−3.4 points	
Schnurr [40]	NR	12 weeks	**PE**	CAPS-5	−15.6 points	**between-group** p < 0.010
			CPT		−13.1 points	
Ford [36]	NR	10 weeks	**TARGET**	CAPS-IV	−39.7 points	NR
			PE		−11.6 points	
Forbes [44]	NR	6 weeks	**CPT**	CAPS*	−27.5 points	NR
			Treatment as usual		−6.8 points	
Gutner [41]	NR	6 weeks	**CPT**	CAPS-IV	−51.8 points	NR
			PE		−50.5 points	
Lloyd [45]	NR	6 weeks	**CPT**	CAPS*	−27.5 points	NR
			Treatment as usual		−6.8 points	
Monson [46]	NR	6 weeks	**CPT**	CAPS-IV	−24.6 points	NR
			Waitlist		−3.1 points	
Resick [42]	NR	6 weeks	**CPT**	CAPS*	−35.7 points	**vs. waitlist** both p < 0.050 **vs. baseline** both p < 0.001
			**PE**		−31.7 points	
			Minimal attention waitlist		−0.6 points	
Resick [43]	NR	~6.15 years	**CPT**	CAPS*	−48.8 points	**between-group** p > 0.050 **vs. posttreatment** p > 0.050
			PE		−48.6 points	
Ehlers [51]	NR	3 months	**CT**	CAPS-2	−48.8 points	**between-group** p < 0.001
			Waitlist		5.8 points	
		6 months	**CT**	CAPS-2	−47.0 points	**vs. baseline** p < 0.0005
					1.8 points	**vs. posttreatment** p > 0.050
Van der Kolk [49]	NR	8 weeks	**EMDR**	CAPS-IV	−36.9 points	**between-group** p = 0.070
			**Placebo**		−26.8 points	

*Note: Specific type of CAPS score was not defined in the study

**Abbreviations**: CAPS – Clinician-Administered PTSD Scale; CBT – Cognitive-behavioral Therapy; PE – Prolonged Exposure Therapy; CPT – Cognitive-processing Therapy; TARGET – Trauma Affect Regulation: Guide for Education and Therapy; CBCT – Cognitive-behavioral conjoint Therapy; NR – Not reported; EMDR – Eye Movement Desensitization and Reprocessing;

Significant post-treatment improvements in PTSD symptoms measured with CAPS scores in waitlist-controlled trials were captured for group cognitive-exposure therapy, PE, CBT, CPT, and CT psychotherapies (24.4, 31.7, 33.4, 35.7, and 48.8 points decreases, respectively; all p<0.001) [33,38,42,51]. RCTs directly comparing PE and CPT noted similar CAPS changes after treatment and long-term observations, but the significance was inconsistently reported [40–43]. Between-group statistical difference was not reported in other studies comparing different types of psychotherapy and psychotherapy with the usual treatment (which were variable, and included waitlist [30], Trauma Affect Regulation: Guide for Education and Therapy (TARGET) [36], and orientation of the therapist [44,45]). Therefore, the superiority of one psychotherapy technique over another was not shown in most of the RCTs with chronic, treatment-resistant, moderate or higher severity PTSD.

Psychotherapy modalities including CT, CPT, PE, and EMDR assessed depression symptoms compared to waitlist arms after treatment. A study by Nacasch et al. showed PE to be similar to treatment as usual (TAU) in BDI changes from baseline at the post-treatment endpoint (12.8- vs. 4.6-point decreases, p = 0.050) [39]. However, numerous studies showed improvement in depression symptoms with psychotherapy compared to waitlist. Ehlers et al. [51] demonstrated the efficacy of 12 weekly CT sessions (an hour each with three monthly boosters) with a significantly greater decrease in BDI from baseline compared to control (23.7 to 10.6 points vs. 23.2 to 19.3 points, p = 0.003). Another 12-week CT trial by Duffy et al. observed a significantly lower BDI score in the CT arm than in waitlist control at post-treatment assessment (22.6 and 32.7 points, respectively; p < 0.001) [50]. A long-term follow-up study of CPT and PE with an average duration 6.2 years found no difference in the BDI during the follow-up period (9.4 and 12.1 points, respectively; p > 0.050 over the follow-up) [43,51]. A study by Duran et al. [37] observed significantly lower BDI scores in CT than the PE arm at post-treatment and 3-month follow-up assessments (11.3 vs. 14.5 points and 12.5 vs. 17.0 points, respectively; p = 0.049). Resick et al. [42] reported significantly lower BDI scores after CPT and PE treatments compared to the Minimal Attention (MA) waitlist (12.7, 16.0, and 22.6 points, respectively; p < 0.001). The improvement in depression symptoms persisted over time in the 6-month follow-up trial of CT (11.2 points, p < 0.001 and p > 0.050 compared to baseline and post-treatment scores). There was no statistical difference between CPT and PE over the ~ 6-year observation [43].

A study conducted by Acarturk et al. among a small sample of Syrian refugees with PTSD demonstrated significant benefits of EMDR therapy in mitigation of depressive symptoms. It was shown that seven 90-min EMDR sessions resulted in significantly lower BDI scores compared to the waitlist control arm at the 7-week post-treatment endpoint (10.15 vs. 20.79, p < 0.010) [47]. A study conducted by Taylor et al. within a sample of 60 patients with chronic PTSD treated with eight 90-min EMDR sessions demonstrated significant decrease in BDI score at the 1-month and 3-month follow-up endpoints compared to the baseline (1-month: 16.4 points vs. 26.4 points; 3-month: 14.4 points vs. 26.4 points; both p < 0.050) [48]. However, the Taylor et al. study lacked between-group differences when comparing effects of EMDR with PE at the 1-month (16.4 points in EMDR vs. 13.0 points in PE, p > 0.050) or 3-month (14.4 points in EMDR vs. 12.7 points in PE, p > 0.050) follow-up endpoints [48].

The evidence around CBT’s effect on depressive symptoms in patients with PTSD is inconsistent. Patients receiving 8 weeks of CBT demonstrated a substantial difference in BDI scores compared to TAU in terrorist-affected patients with PTSD immediately after treatment (3.2 vs. 11.3 points, p = 0.004) and 3-month assessments (6.4 vs. 11.0 points, p = 0.003) [35]. Akbarian et al. showed significant improvement in BDI scores after 10-session CBT treatment compared to waitlist controls (27.5 and 21.0 points decrease, respectively; p < 0.050) [29]. However, Fecteau et al. [33] observed insignificantly different BDI score changes between CBT and waitlist at post-treatment assessment (decrease from 26.3 to 20.1 points in CBT and 27.9 to 24.7 points in waitlist). A significant change in BDI score from baseline was captured only after the 6-month follow-up in CBT (from 26.3 to 15.9 points, p < 0.050) [33].

Significant improvement in functional impairment was captured in only two 12-week waitlist-controlled CT trials. Ehlers et al. [51] reported significant SDS score improvement from baseline in the CT arm compared to waitlist (7.6 to 3.0 points vs. 6.7 to 6.3 points, respectively, both p < 0.0005). Post-treatment scores in CT patients did not change over the 6-month follow-up [51]. Duffy et al. [50] showed a statistical difference between post-treatment SDS scores in CT patients compared to waitlist control (5.3 and 7.4 point reduction in CT and waitlist, respectively; p = 0.045).

#### FDA-approved medications.

This SLR includes eight RCTs with FDA-approved PTSD medications; four each with paroxetine or sertraline. Of the paroxetine trials, three included a placebo control arm [52–54] and one a mirtazapine control arm [55]. Of the sertraline studies, two were placebo-controlled [56,57], one compared efficacy of sertraline vs. sertraline in combination with psychotherapy [58], and one compared sertraline with placebo and venlafaxine [59]. FDA-approved PTSD medication trials that assessed clinical efficacy using CAPS score had a follow-up duration of 8–24 weeks. Results of CAPS score changes after treatment with paroxetine and sertraline are summarized in Table 5.

**Table 5:** Summary of post-treatment CAPS score changes from clinical trials with FDA-approved medications.

Author	Study Phase	Time Endpoint	Intervention	CAPS Type	CAPS Change	P-value
Marshall [53]	NR	10 weeks	**Paroxetine**	CAPS-2	−27.2 points	NR
			Placebo		−21.4 points	
Schneier [54]	NR	22 weeks	**Paroxetine + PE**	CAPS-IV	−42.2 points	**between-group** p > 0.050
			Placebo + PE		−37.5 points	
Marshall [52]	NR	12 weeks	**Paroxetine 20 mg**	CAPS-2	−39.6 points	**between-group** **vs. placebo** both p < 0.001
			**Paroxetine 40 mg**		−37.9 points	
			Placebo		−25.3 points	
Seo [55]	NR	8 weeks	**Paroxetine**	CAPS-2	−39.6 points	**vs. baseline** both p < 0.001 **between-group** p = 0.691
			Mirtazapine		−38.1 points	
Rauch [58]	NR	24 weeks	**Sertraline + EMM**	CAPS-IV	−33.8 points	**vs. baseline** both p < 0.001 **vs. placebo** both p > 0.050
			**Sertraline + PE**		−32.7 points	
			Placebo + PE		−29.4 points	
Zohar [56]	NR	10 weeks	**Sertraline**	CAPS-2	−18.7 points	**between-group** p = 0.530
			Placebo		−13.5 points	
Davidson [57]	NR	12 weeks	**Sertraline**	CAPS-2	−33.0 points	**vs. baseline** p = 0.040
			Placebo		−26.2 points	
Davidson [59]	NR	12 weeks	**Sertraline**	CAPS-2	−39.4 points	**vs. venlafaxine** p = 0.494 **vs. placebo** p = 0.081
			Venlafaxine		−41.5 points	
			Placebo		−34.2 points	

**Abbreviations**: CAPS – Clinician-Administered PTSD Scale; PE – Prolonged Exposure therapy; NR – Not reported; EMM – Enhanced Medication Management

**Note: p > 0.050 indicates lack of significant statistical difference**

There is inconsistent evidence related to the efficacy of FDA-approved medications in improving symptoms of chronic, treatment-resistant, moderate or higher severity PTSD. Marshall et al. [52] reported significant CAPS improvement after 12-week treatment with 20 mg and 40 mg paroxetine compared to placebo. Paroxetine was not superior to mirtazapine as the changes in CAPS-2 scores were not statistically different between study arms (p = 0.691) [55]. An RCT by Schneier et al. [54] did not observe significant changes in CAPS scores at the 22-week post-treatment endpoint between paroxetine with PE and placebo with PE arms in World Trade Center survivors. Zohar et al. [56] failed to show statistical difference in CAPS score changes after 10 weeks of sertraline treatment compared to placebo. Although Davidson et al. [59] reported higher CAPS-2 score decreases from baseline in sertraline and placebo arms after 12-weeks of treatment, the results were not significantly different. Other sertraline trials observed a significant CAPS score reduction from baseline (~33 points) but did not report between-group statistics compared to placebo or active controls [57,58]. The efficacy of sertraline monotherapy in reducing PTSD symptom severity was not different from PE, sertraline-augmented PE, or venlafaxine treatments [58,59].

A few RCTs reported statistical differences in clinical scores other than CAPS among FDA-approved medications for PTSD. A study by Seo et al. [55] in severe Korean patients with PTSD demonstrated a significant decrease in BDI-II score from baseline in the 60 mg paroxetine arm (9.7 points reduction, p < 0.001). A study conducted by Marshall et al. [52] observed a significantly greater decrease in SDS score after 12-week treatment with 20 mg and 40 mg paroxetine compared to placebo (7.0 and 6.4 vs. 4.5 point reduction from baseline, respectively; both p < 0.020) [52]. Davidson et al. [59] noted a higher but not statistically significant SDS score improvement from baseline after 12-week sertraline and placebo treatment (8.2- vs. 6.5-point reduction, respectively; p = 0.068).

#### Off-label medications.

A total of 33 trials evaluated off-label medication efficacy for treatment of PTSD via the most relevant clinical scores in patients with chronic, treatment-resistant, moderate or higher severity PTSD. Specifically, there were four prazosin [60–63], three D-cycloserine augmented with virtual reality exposure (VRE) or PE therapy [64–66], three risperidone [67–69], three fluoxetine [49,70,71], one mirtazapine [55], two topiramate [72,73], two venlafaxine [59,74], two propranolol [75,76], and two eszopiclone RCTs [77,78]. Additionally, there was a single study captured for each of the following off-label medications used for PTSD treatment: ketamine [79], divalproex [80], nabilone [81], ganaxolone [82], olanzapine [83], cyclobenzaprine [84], nefazodone [85], mifepristone [86], and tiagabine [87]. Length of follow-up across off-label medication trials that evaluated clinical efficacy via CAPS score changes ranged from 3 to 26 weeks. CAPS score changes after treatment with off-label medications for PTSD are summarized in Table 6.

**Table 6:** Post-treatment CAPS score changes from clinical trials with off-label PTSD medications.

Author	Study Phase	Time Endpoint	Intervention	CAPS Type	CAPS Change	P-value
Abdallah [79]	NR	8 weeks	**Low-dose ketamine**	CAPS-5	**vs. placebo** −8.4 points	p = 0.030
			**Standard-dose ketamine**		**vs. low-dose** 2.7 points	p = 0.430
			Placebo		**vs. standard dose** 5.7 points	p = 0.130
Davis [80]	NR	8 weeks	**Divalproex**	CAPS^*^	−15.1 points	**between-group** p ≥ 0.050
			Placebo		−16.5 points	
Jetly [81]	NR	7 weeks	**Nabilone**	CAPS-IV^*^	−3.6 points	**between-group** p = 0.030
			Placebo		−1.0 points	
Rasmusson [82]	Phase 2	6 weeks	**Ganaxolone**	CAPS-IV^*^	−17.6 points	**between-group** p = 0.550
			Placebo		−15.1 points	
van der Kolk [49]	NR	8 weeks	**Fluoxetine**	CAPS-IV^*^	−31.0 points	**Fluoxetine vs. EMDR** p = 0.130 **Fluoxetine vs. placebo** p = 0.610
			EMDR		−36.9 points	
			Placebo		−26.8 points	
Martenyi [70]	NR	12 weeks	**Fluoxetine**	CAPS^*^	−31.1 points	**between-group** p < 0.001
			Placebo		−16.1 points	
Martenyi [71]	NR	12 weeks	**Fluoxetine 20 mg**	CAPS^*^	−42.9 points	**between-group** p = 0.151
			**Fluoxetine 40 mg**		−42.8 points	
			Placebo		−36.6 points	
Carey [83]	NR	8 weeks	**Olanzapine**	CAPS^*^	−45.8 points	**between-group** p = 0.018
			Placebo		−19.3 points	
Raskind [61]	NR	9 weeks	**Prazosin**	CAPS^*^	−21.8 points	**between-group** p < 0.010
			Placebo		2.9 points	
Raskind [62]	NR	8 weeks	**Prazosin**	CAPS^*^	−13.0 points	**between-group** p = 0.300
			Placebo		−7.0 points	
Raskind [63]	NR	15 weeks	**Prazosin**	CAPS^*^	−25.1 points	**between-group** p = 0.020
			Placebo		−13.8 points	
Raskind [60]	NR	26 weeks	**Prazosin**	CAPS-IV^*^	−14.4 points	**between-group** p = 0.481
			Placebo		−17.9 points	
Difede [65]	NR	12 weeks	**D-Cycloserine + VRE**	CAPS-IV^*^	−49.2 points	**between-group** p = 0.131
			Placebo + VRE		−32.9 points	
Rothbaum [64]	NR	5 weeks	**D-Cycloserine + VRE**	CAPS^*^	−19.4 points	**between-group** p = 0.320 **vs. baseline** p < 0.001
			Placebo + VRE		−18.8 points	
de Kleine [66]	NR	8-10 weeks	**D-Cycloserine + PE**	CAPS-1	−27.4 points	**between-group** p = 0.620
			Placebo + PE		−20.2 points	
Sullivan [84]	Phase 2	12 weeks	**High-dose cyclobenzaprine**	CAPS-5	−19.1 points	**High-dose vs placebo** p = 0.037 **Low-dose vs. placebo** p = 0.172
			**Low-dose cyclobenzaprine**		−17.2 points	
			Placebo		−14.6 points	
Seo [55]	NR	8 weeks	**Mirtazapine**	CAPS-2	−38.1 points	**vs. baseline** both p < 0.001 **between-group** p = 0.691
			Paroxetine		−39.6 points	
Davis [85]	NR	12 weeks	**Nefazodone**	CAPS^*^	−19.1 points	**between-group** p = 0.040
			Placebo		−13.5 points	
Brunet [75]	NR	6 weeks	**Propranolol + TMR**	CAPS^*^	−27.0 points	**between-group** p < 0.010
			Placebo + TMR		−11.7 points	
Brunet [76]	NR	8 weeks	**Propranolol + TMR**	CAPS^*^	−28.9 points	**between-group** p = 0.034
			Placebo + TMR		−17.4 points	
Bartzokis [67]	NR	16 weeks	**Risperidone**	CAPS^*^	−14.3 points	**between-group** p < 0.050 **vs. baseline** p < 0.001
			Placebo		−4.6 points	
Krystal [68]	NR	24 weeks	**Risperidone**	CAPS^*^	−13.8 points	**between-group** p = 0.120 **vs. baseline** p < 0.001
			Placebo		−11.0 points	
Padala [69]	NR	12 weeks	**Risperidone**	CAPS^*^	−23.9 points^†^	**vs. baseline** p < 0.050
			Placebo		−10.6 points^†^	
Davidson [87]	NR	12 weeks	**Tiagabine**	CAPS^*^	−30.7 points	**between-group** p = 0.850
			Placebo		−30.2 points	
Yeh [72]	NR	12 weeks	**Topiramate**	CAPS^*^	−48.4 points	**between-group** p = 0.490
			Placebo		−30.4 points	
Monga [73]	NR	12 weeks	**Topiramate**	CAPS^*^	−27.4 points	**between-group**^1^ p = 0.310
			Placebo		−24.2 points	
Davidson [74]	NR	24 weeks	**Venlafaxine ER**	CAPS-SX _17_	−51.8 points	**between-group** p = 0.006
			Placebo		−44.8 points	
Davidson [59]	NR	12 weeks	**Venlafaxine ER**	CAPS-SX_17_	−41.5 points	**vs. placebo** p = 0.015 **vs. sertraline** p = 0.494
			Sertraline		−39.4 points	
			Placebo		−34.2 points	
Golier [86]	Phase 2a	12 weeks	**Mifepristone**	CAPS^*^	−15.2 points	**between-group** p = 0.570
			Placebo		−18.1 points	
Pollack [77]	NR	3 weeks	**Eszopiclone**	CAPS^*^	−21.2 points	**between-group** p = 0.003
			Placebo		−0.6 points	
Dowd [78]	NR	12 weeks	**Eszopiclone**	CAPS^*^	−25.0 points	**between-group** p = 0.700 **vs. baseline** p = 0.002
			Placebo		−23.0 points	

* **Note:** Specific type of CAPS score was not defined in the study

^1^The statistical difference between percentual change from baseline (not means)

† Extrapolated data

**Abbreviations**: CAPS – Clinician-Administered PTSD Scale; EMDR – Eye Movement Desensitization and Reprocessing; VRE – Virtual Reality Exposure; TMR – Traumatic Memory Reactivation; ER – Extended Release

Off-label medications used for PTSD treatment that consistently showed significant improvement in the post-treatment CAPS scores from baseline compared to placebo were propranolol (with TMR), olanzapine, venlafaxine ER, nefazodone, and nabilone [59,74–76,81,83,85]. Administration of propranolol prior to TMR sessions showed a substantial decrease in baseline CAPS values compared to placebo in two RCTs [75,76]. Two venlafaxine ER trials using 300 mg over 12 and 24 weeks also demonstrated significant CAPS-SX_17_ decreases from baseline compared to placebo [59,74]. Placebo-controlled trials of olanzapine, nefazodone, and nabilone reported significantly greater post-treatment CAPS changes from baseline than comparator arms [81,83,85].

Dose-dependent efficacy was observed for cyclobenzaprine, fluoxetine, and ketamine. After 12-week treatment with cyclobenzaprine, only a high-dose arm (5.6 mg per day) led to a significantly greater CAPS-5 score reduction from baseline, while a lower change in a low-dose arm (2.8 mg per day) was not statistically different compared to the control arm [84]. Fluoxetine trials also reported better treatment response in higher doses (80 mg daily). High-dose fluoxetine had greater CAPS improvements than the placebo arm at post-treatment and at long-term follow-up (12-weeks from treatment completion). Lower fluoxetine doses (20–60 mg daily) reported similar ranges of CAPS score changes as 80 mg treatment but without between-group differences compared to placebo [49,70,71]. Unlike cyclobenzaprine and fluoxetine, low-dose ketamine (0.2 mg/kg) appeared superior to the standard dose (0.5 mg/kg) in reducing PTSD symptoms severity after 4 weeks of treatment [79].

Besides the dose-dependent effectiveness of cyclobenzaprine, fluoxetine, and ketamine, there was heterogeneous evidence for prazosin, risperidone, and eszopiclone in placebo-controlled trials. Post-treatment CAPS decreases ranged from 13.0 to 25.1 points for prazosin, 13.8 to 23.9 points for risperidone, and 21.2 to 25.0 points for eszopiclone; however, the between-group statistical difference was inconsistently reported [60–63,67–69,77,78].

Off-label medications used for PTSD treatment that consistently failed to reach significantly greater CAPS score decreases at post-treatment endpoints compared to placebo arms among captured trials were ganaxolone, tiagabine, mifepristone and topiramate studies [72,73,82,86,87]. Direct comparisons between off-label medications and other treatments did not observe the superiority of one therapy over another.

Only a few trials reported changes in clinical scores related to depression symptoms (BDI), functional impairment (SDS), and dissociative symptoms (DES). Statistically significant improvement in SDS scores from baseline was observed following olanzapine and venlafaxine ER treatments compared to placebo [59,74,83]. The post-treatment changes in BDI [66,72,86] BDI-II [49,55], SDS [84,87], and/or DES [71] scales after other off-label treatments did not reach statistically significant difference compared to control arms.

BDI post-treatment improvement from baseline was not statistically significant for topiramate compared to placebo (8.5 vs. 3.9 points decreases, respectively; p = 0.720) [72]. Fluoxetine also showed similar post-treatment BDI scores to placebo (13.0 and 14.4 points, respectively; p = 0.940) and EMDR (9.10 points, p = 0.080). The score remained stable during the 6-month period after fluoxetine treatment but decreased in the EMDR arm (14.0 and 5.25 points, respectively; p < 0.001) [49]. Although mirtazapine led to a statistically significant decrease in BDI score after treatment (9.0 point decrease from baseline, p < 0.001), there was no statistical difference compared to the change in the paroxetine control arm (9.7 points decrease, p = 0.441) [55]. A phase IIa, placebo-controlled, double-blind study demonstrated similar effects of mifepristone and placebo in diminishing depression symptoms related to PTSD with decreases in BDI scores at 4-week (3.5 and 4.5 points, respectively; p = 0.600) and 12-week (2.3 and 5.1 points, respectively; p = 0.200) endpoints [86]. D-cycloserine with PE showed a similar BDI decrease as the placebo with PE arm (8.5 and 6.6 points, respectively; p = 0.730) [66].

Olanzapine was associated with significantly lower SDS scores after treatment compared to placebo (10.6 vs. 20.6 points, respectively; p = 0.004) [83]. Venlafaxine ER also led to a substantial improvement in functional impairment; after 12 and 24 weeks of treatment, a statistically greater decrease in SDS scores from baseline was seen after treatment with venlafaxine ER compared to placebo (8.5 vs. 6.5 and 10.1 vs. 8.0 points, respectively; p-values 0.025 and 0.030, respectively). However, venlafaxine ER failed to reach significance in SDS score change compared to sertraline after 12 weeks (8.5 vs. 8.2 points decrease, respectively; p = 0.683) [59,74]. Other off-label medications which did not show statistically significant difference in SDS score changes from baseline compared to placebo were cyclobenzaprine (scores not reported, p = 0.080) and tiagabine (−5.5 points for tiagabine and −5.9 for placebo; p = 0.740) [84,87].

Improvement in dissociative symptoms measured with the DES scale was reported in only one fluoxetine trial. The study observed insignificant post-treatment changes from baseline in 20 mg and 40 mg fluoxetine arms compared to placebo (scores not reported, p > 0.050) [71].

#### Key summary.

All MDMA-AT RCTs reported significant CAPS improvements after treatment compared to placebo and low-dose active controls. However, between-group significances in CAPS score changes were inconsistently reported among placebo-controlled trials of FDA-approved and most off-label medications used for PTSD treatment, including combination treatment with psychotherapy. Statistically greater CAPS improvement was observed in propranolol (with TMR), olanzapine, venlafaxine ER, nefazodone, and nabilone studies, while cyclobenzaprine, fluoxetine, and ketamine showed dose-dependent efficacy. However, evidence of efficacy in reducing PTSD symptoms measured with CAPS in patients with chronic, treatment-resistant, moderate or higher severity PTSD was captured in only a few trials per medication. Although most psychotherapy trials did not report between-group statistics, CBT modalities, PE, CPT, and CT showed consistently greater CAPS changes from baseline than waitlist controls. Direct comparisons between medications and medications with psychotherapies did not show the superiority of one treatment option over another.

BDI scores and statistics of BDI changes were rarely reported. MDMA-AT at a dose of 120–180 mg or 125–187.5 mg showed significantly greater improvement in depression symptoms compared to placebo or 30 mg MDMA-AT controls. However, the significance was not achieved compared to 40 mg MDMA-AT arms. None of the medications used for PTSD treatment (FDA-approved or off-label) had a statistically different BDI score change from baseline compared to placebo controls. Among psychotherapy trials, BDI score improvement compared to waitlist controls was observed in CT, CPT, and PE. While one study showed BDI score improvement with EMDR compared to the waitlist, another study showed an insignificant difference compared to placebo. CBT was the only psychotherapy showing superiority compared to treatment as usual in terms of BDI scores. Looking at comparative psychotherapy types, EMDR was shown to be superior to fluoxetine, and CT showed a greater reduction in depression symptoms than PE.

The clinical efficacy of relevant PTSD treatments is summarized and presented in Table 7. Green-colored cells are used for studies that report the significant between-arm difference, red-colored cells represent those that did not find the significant between-arm difference, and gray-colored cells were used for studies that did not assess and report between-arm difference (abbreviated ‘NR’).

**Table 7:** Summary of clinical efficacy of relevant PTSD treatments.

Author	Intervention	Comparator	Significant between-arm CAPS improvement	Significant between-arm BDI improvement	Significant between-arm SDS improvement	Significant between-arm DES improvement
**MDMA-AT**						
Mitchell^1^ [15]	120-180 mg MDMA-AT^†^	Placebo-AT	✓	✓	✓	NR
Mitchell [16]	120-180 mg MDMA-AT^‡^	Placebo-AT	✓	NR	✓	NR
van der Kolk^2^ [28]	120-180 mg MDMA-AT^†^	Placebo-AT	✓	NR	NR	NR
Mithoefer [23]	125-187.5 mg MDMA-AT	Placebo-AT	✓	NR	NR	NR
Mithoefer [24]	75-187.5 mg MDMA-AT	30-45 mg MDMA-AT	✓	✓	NR	✓
Oehen [26]	125-187.5 mg MDMA-AT	25-37.5 mg MDMA-AT	✓	NR	NR	NR
Ot’alora^1^ [25]	125-187.5 mg MDMA-AT	40-60 mg MDMA-AT	✓	✗	NR	✗
**FDA-Approved Medications**						
Davidson [57]	Sertraline	Placebo	✓	NR	NR	NR
Zohar [56]	Sertraline	Placebo		NR	NR	NR
Rauch^3^ [58]	Sertraline + /- PE	Placebo + PE	✗	NR	NR	NR
Davidson [59]	Sertraline	Placebo	✗	NR	NR	NR
	Sertraline	Venlafaxine ER	✗	NR	✗	NR
Marshall [52]	20 mg Paroxetine	Placebo	✓	NR	✓	NR
	40 mg Paroxetine	Placebo	✓	NR	✓	NR
Schneier [54]	Paroxetine + PE	Placebo + PE	✗	NR	NR	NR
Seo [55]	Paroxetine	Mirtazapine	✗	✗	NR	NR
**Off-Label Medications**						
Abdallah [79]	0.2 mg/kg Ketamine	Placebo	x	NR	NR	NR
	0.5 mg/kg Ketamine	0.2 mg/kg Ketamine or Placebo	✗	NR	NR	NR
Brunet [75]	Propranolol + TMR	Placebo + TMR	✓	NR	NR	NR
Brunet [76]	Propranolol + TMR	Placebo + TMR	✓	NR	NR	NR
Carey [83]	Olanzapine	Placebo	✓	NR	✓	NR
Davidson [59,74]	Venlafaxine ER	Placebo	✓	NR	✓	NR
Davidson [87]	Tiagabine	Placebo	✗	NR	✗	NR
Davis [85]	Nefazodone	Placebo	✓	NR	NR	NR
Jetly [81]	Nabilone	Placebo	✓	NR	NR	NR
Davis [80]	Divalproex	Placebo	✗	NR	NR	NR
Krystal [68]	Risperidone	Placebo	✗	NR	NR	NR
Bartzokis [67]	Risperidone	Placebo	✓	NR	NR	NR
Raskind [61]	Prazosin	Placebo	✓	NR	NR	NR
Raskind [62]	Prazosin	Placebo	✗	NR	NR	NR
Raskind [63]	Prazosin	Placebo	✓	NR	NR	NR
Raskind [60]	Prazosin	Placebo	✗	NR	NR	NR
Rasmusson [82]	Ganaxolone	Placebo	✗	NR	NR	NR
Sullivan [84]	5.6 mg Cyclobenzaprine	Placebo	✓	NR	✗	NR
	2.8 mg Cyclobenzaprine	Placebo	✗	NR	NR	NR
Yeh [72]	Topiramate	Placebo	✗	✗	NR	NR
Monga [73]	Topiramate	Placebo	✗	NR	NR	NR
Golier [86]	Mifepristone	Placebo	✗	✗	NR	NR
	Mifepristone^5^	Placebo^5^	✗	✗	NR	NR
Pollack [77]	Eszopiclone	Placebo	✓	NR	NR	NR
Dowd [78]	Eszopiclone	Placebo	✗	NR	NR	NR
van der Kolk [49]	Fluoxetine	Placebo	✗	✗	NR	NR
		EMDR	✗	✗	NR	NR
Martenyi [71]	20 mg Fluoxetine	Placebo	✗	NR	NR	✗
	40 mg Fluoxetine	Placebo	✗	NR	NR	✗
Martenyi [70]	Fluoxetine	Placebo	✓	NR	NR	NR
de Kleine [66]	D-cycloserine + PE	Placebo + PE	✗	✗	NR	NR
Difede [65]	D-Cycloserine + VRE	Placebo + VRE	✗	NR	NR	NR
	D-Cycloserine + VRE^4^	Placebo + VRE^4^	✓	NR	NR	NR
Rothbaum [64]	D-Cycloserine + VRE	Placebo + VRE	✗	NR	NR	NR
	D-Cycloserine + VRE^5^	Placebo + VRE^5^	✗	NR	NR	NR
**Psychotherapies**						
Fecteau [33]	CBT	Waitlist	✓	✗	NR	NR
Akbarian [29]	CBT	Waitlist	NR	✓	NR	NR
Bryant [35]	CBT	Treatment as usual	NR	✓	NR	NR
	CBT^7^	Treatment as usual^7^	NR	✓	NR	NR
Castillo [38]	Group Cognitive/ Exposure Therapy	Waitlist	✓	NR	NR	NR
Resick [42]	CPT or PE	MA Waitlist	✓	✓	NR	NR
Nacasch [39]	PE	Treatment as usual	NR	✗	NR	NR
Schnurr [40]	PE	CPT	✓	✗	NR	NR
	PE^7^	CPT^7^	✓	✗	NR	NR
	PE^4^	CPT^4^	✗	✗	NR	NR
Resick^6^ [43]	PE	CPT	✗	✗	NR	NR
Acarturk [47]	EMDR	Waitlist	NR	✓	NR	NR
Taylor [48]	EMDR	PE	NR	✗	NR	NR
	EMDR^7^	PE	NR	✗	NR	NR
van der Kolk [49]	EMDR	Placebo	✗	✗	NR	NR
	EMDR^4^	60 mg Fluoxetine^4^	✓	✓	NR	NR
Ehlers [51]	CT	Waitlist	✓	✓	✓	NR
Duffy [50]	CT	Waitlist	NR	✓	✓	NR
Duran^1^ [37]	CT	PE	NR	✓	NR	NR
	CT^7^	PE^7^	NR	✓	NR	NR

* **Note:** All results reported at post-treatment endpoints if not stated otherwise

† The 120–180 mg was a split dose of 80 + 40 mg for the first session and 120 + 60 mg in the second and third experimental sessions. Six participants chose either not to take the supplemental dose (n = 3, 1 MDMA) or not to escalate to the 120 mg dose (n = 3, 2 MDMA) in a total of six experimental sessions (2.3% of the total sessions across the study) [15].

‡ *Three participants did not undergo dose escalation in sessions 2 and 3 [16].*

^1^ *Completers population*

^2^ *Subgroups with borderline/diagnosed alexithymia and low self-compassion scale*

^3^ *Two intervention arms, one with PE and one without PE*

^4^ *6-month follow-up*

^5^ *12-month follow-up*

^6^ *Long-term follow-up*

^7^ *3-month follow-up*

***Abbreviations:*** *CAPS – Clinician-Administered PTSD Scale; BDI – Beck Depression Inventory; SDS – Sheehan Disability Scale; DES – Dissociative Experience Scale; MDMA – 3,4-Methylenedioxymethamphetamine; NR – Not reported; PE – Prolonged exposure; TMR – Traumatic Memory Reactivation; ER – Extended Release; VRE – Virtual reality exposure; CBT – Cognitive behavioral therapy; Cognitive processing therapy – CPT; MA Waitlist – Minimal Attention Waitlist; CT – Cognitive therapy; EMDR – Eye movement desensitization and reprocessing*

### Disease course measures

#### MDMA-AT.

There were three phase II trials [23–25] and two phase III studies [15,16] that demonstrated disease course measures after MDMA-AT treatment for PTSD. The rates of clinical response, loss of PTSD diagnosis and disease remission are presented in Table 8.

**Table 8:** Summary of post-treatment disease course measures from MDMA-AT publications.

Author	Year	Phase	PTSD Treatment	Rate (%)	Outcome Definition
**Clinical Response**					
Mitchell [15]	2021	Phase 3	120-180 mg^*^ MDMA-AT	90.7%	≥10-point decrease on CAPS-5
			Placebo + AT	84.3%	
Mitchell [16]	2023	Phase 3	120-180 mg^†^ MDMA-AT	86.5%	≥10-point decrease on CAPS-5
			Placebo + AT	69.0%	
Mithoefer [23]	2011	Phase 2	125-187.5 mg MDMA-AT	83.3%	≥30% post-treatment CAPS-IV score reduction
			Placebo + AT	25.0%	
Mithoefer [24]	2018	Phase 2	125 −187.5 mg MDMA-AT	67.0%	
			75-112.5 mg MDMA-AT	100.0%	
			30-45 mg MDMA-AT	29.0%	
Ot’alora [25]	2018	Phase 2	125-187.5 mg MDMA-AT	50.0%	
			100-150 mg MDMA-AT	55.6%	
			40-60 mg MDMA-AT	16.7%	
**Loss of Diagnosis**					
Mitchell [15]	2021	Phase 3	120-180 mg MDMA-AT^*^	67.0%	Specific diagnostic measure on the CAPS-5
			Placebo + AT	32.0%	
Mitchell [16]	2023	Phase 3	120-180 mg MDMA-AT^†^	71.2%	Based on the DSM-5 criteria for PTSD
			Placebo + AT	47.6%	
Mithoefer [23]	2011	Phase 2	125-187.5 mg MDMA-AT	83.3%	Based on the DSM-IV criteria for PTSD
			Placebo + AT	25.0%	
Mithoefer [24]	2018	Phase 2	125-187.5 mg MDMA-AT	58.0%	Based on the DSM-IV criteria for PTSD measured with CAPS-IV
			75-112.5 mg MDMA-AT	86.0%	
			30-45 mg MDMA-AT	29.0%	
Ot’alora [25]	2018	Phase 2	125-187.5 mg MDMA-AT	41.7%	Based on the DSM-IV criteria for PTSD measured with CAPS-IV
			100-150 mg MDMA-AT	44.4%	
			40-60 mg MDMA-AT	33.3%	
**Remission**					
Mitchell [15]	2021	Phase 3	120-180 mg MDMA-AT^*^	33.0%	CAPS-5 score ≤11 points and loss of PTSD diagnosis
			Placebo + AT	5.0%	
Mitchell [16]	2023	Phase 3	120-180 mg^†^ MDMA-AT	46.2%	
			Placebo + AT	21.4%	

* The 120–180 mg was a split dose of 80 + 40 mg for the first session and 120 + 60 mg in the second and third experimental sessions. Six participants chose either not to take the supplemental dose (n = 3, 1 MDMA) or not to escalate to the 120 mg dose (n = 3, 2 MDMA) in a total of six experimental sessions (2.3% of the total sessions across the study) [15].

† *Three participants did not undergo dose escalation in sessions 2 and 3* [16].

Clinical response (defined as either > 30% [23,24] or ≥30.0% [25] post-treatment CAPS-IV score reduction was reported in three phase II MDMA-AT trials, with a response rate between 16.7% (40 mg) and 100% (75 mg) for the MDMA-AT arms [23–25]. Clinical response (defined as ≥10-point reduction in CAPS score) was also seen in the two phase III trials, with response rates of 86.5 to 90.7% in the MDMA-AT arms [15,16].

Loss of diagnosis (defined as not meeting diagnostic criteria for PTSD at study endpoint) rates was 71.2% [16] and 67.0% [15] in the two Phase III studies (120–180 mg). For the Phase II trials, loss of diagnosis in the MDMA-AT arms ranged from 29% (30 mg) to 86.0% (75 mg) [24].

Remission rate in the two Phase III trials, defined as a CAPS-5 score ≤11 points and loss of PTSD diagnosis, were 33.0% among patients with severe PTSD [15] and 46.2% among patients with moderate or higher severity PTSD [16].

Relapse rate (defined as not meeting diagnostic criteria for PTSD after treatment but relapsed during the follow-up) was reported in the phase II Mithoefer et al. [24] study as 2 participants (8.3%) at treatment exit who did not meet PTSD criteria based on CAPS-IV and relapsed at the 12-month follow-up.

#### Psychotherapies.

There were three RCTs [33,46,88] that evaluated clinical response rate and 11 studies [32–34,36,38,40,42,46,48,49,88] that assessed rates of PTSD loss of diagnosis after treatment by various psychotherapy modalities. The rates of clinical response and loss of PTSD diagnosis from these studies are presented in Table 9.

**Table 9:** Summary of post-treatment disease course measures from clinical trials with psychotherapies.

Author	Year	PTSD Treatment	Rate (%)	Outcome Definition
**Clinical Response**				
Fecteau [33]	1999	CBT	80.0%	≥11.09 points decrease in CAPS score
		Waitlist	20.0%	
Monson [46]	2006	CPT	47.0%	≥12.0 points decrease in CAPS score
		Waitlist	30.0%	
Ter Heide [88]	2016	EMDR	40.6%	≥10 points decrease in CAPS score
		Stabilisation as usual	41.9%	
**Loss of Diagnosis**				
Fecteau [33]	1999	CBT	50.0%	Based on the DSM-IV criteria for PTSD
		Waitlist	0.0%	
Beck [34]	2009	Group CBT	88.3%	Based on the DSM-IV criteria for PTSD
		Minimal Contact Group	31.3%	
Castillo [38]	2016	CET	51.9%	Total CAPS score ≤45.0
		Waitlist	NR	
Ford [36]	2018	TARGET	36.0%	Based on the DSM-IV criteria for PTSD Intent-to-treat
		PE	21.0%	
Monson [32]	2012	CBCT	81.0%	Met criteria for PTSD and a total severity score lower than 45 on the CAPS
		Waitlist	21.0%	
Monson [46]	2006	CPT	40.0%	Based on the DSM-IV criteria for PTSD
		Waitlist	3.0%	
Resick [42]	2002	CPT	53.0%	Based on the CAPS score, using only symptom but not time criteria
		PE	53.0%	
		MA Waitlist	2.2%	
Schnurr [40]	2022	PE	40.4%	Treatment response, no longer meeting DSM-5 PTSD criteria, and CAPS-5 < 25.0
		CPT	28.2%	
Ter Heide [88]	2016	EMDR	19.0%	Based on the DSM-IV criteria for PTSD
		Stabilisation as usual	29.0%	
van der Kolk [49]	2007	EMDR	76.0%	Based on the DSM-IV criteria for PTSD
		Placebo	59%	
Taylor [48]	2003	EMDR	60.7%	Based on the DSM–IV criteria for PTSD
		PE	87.0%	

***Abbreviations*** *: CAPS – Clinician-Administered PTSD Scale; CBT – Cognitive-behavioral Therapy; CPT – Cognitive-processing Therapy; EMDR – Eye Movement Desensitization and Reprocessing; DSM – Diagnostic and Statistical Manual of Mental Disorders; CET – Cognitive/Exposure Therapy; TARGET – Trauma Affect Regulation: Guide for Education and Therapy; PE – Prolonged Exposure therapy; CBCT – Cognitive-behavioral conjoint Therapy; MA – Minimal Attention*

Reported clinical response rate was 80.0% in patients treated with CBT [33], 47.0% in patients who received CPT [46], and 40.6% in patients treated with EMDR [88]. Monson et al. [46] observed 10.0% of patients who had PTSD progression after receiving CPT [46]. Bryant et al. [35] reported a high end-state functioning rate, defined as a percentage of patients with CAPS <19.0 points and BDI < 10.0 points, in 75.0% of CBT arm participants

Loss of PTSD diagnosis was the most frequently reported outcome regarding PTSD course after psychotherapy intervention. Post-treatment rates among studies were 36.0–88.3% for CBT [32–34,36,38], 21.0–53.0% for PE [36,40,42,48], 19.0–76.0% for EMDR [48,49,88], and 28.2–53.0% for CPT arms [40,42,46].

#### FDA-approved medications.

Following the FDA’s approval of medications for the treatment of PTSD, there were seven clinical trials [52,54,55,57,58,89,90] that evaluated the course of PTSD utilizing clinical outcomes. The course of PTSD among these studies are presented in Table 10.

**Table 10:** Summary of clinical studies of FDA-approved medications for PTSD: Disease course outcomes.

Author	Year	PTSD Treatment	Rate (%)	Outcome Definition
**Clinical Response**				
Marshall [52]	2001	20 mg Paroxetine	62.6%	CGI Scales 1 or 2
		40 mg Paroxetine	56.6%	
		Placebo	36.6%	
Seo [55]	2010	Paroxetine	85.0%	≥30.0% reduction in CAPS-2
		Mirtazapine	70.0%	
Li [89]	2017	Sertraline	49.0%	≥30.0% reduction in IES-R total score
		Placebo	6.0%	
Panahi [90]	2011	Sertraline	40.0%	IES-R score reduction by ≥30.0% and CGI score of 1 or 2
		Placebo	6.0%	
Davidson [57]	2001	Sertraline	66.5%	≥30.0% decrease in the CAPS score or CGI rating of 1 or 2
		Placebo	40.8%	
**Remission**				
Schneier [54]	2012	Paroxetine	45.5%	CAPS score of ≥20.0 and a CGI score of 1
		Placebo	45.5%	
Rauch [58]	2019	Sertraline + EMM	39.4%	CAPS score ≤35.0 points
		Sertraline + PE	37.7%	
		Placebo + PE	20.9%	

***Abbreviations:*** *CGI – Clinical Global Impressions; CAPS – Clinician-Administered PTSD Scale; IES-R – Impact of Event Scale–Revised; EMM – Enhanced Medication Management; PE – Prolonged Exposure therapy*

None of the captured RCTs reported loss of PTSD diagnosis rates. Treatment response and PTSD remission rates were predominantly defined in RCTs using Clinical Global Impressions (CGI) scales or CAPS scores. Treatment response in patients with PTSD treated with paroxetine ranged from 56.6%−85.0%, while in sertraline-treated patients, response ranged from 40.0–66.5%. Two RCTs [54,58] observed remission rates among patients with PTSD treated with FDA-approved medications; 45.5% in paroxetine-treated patients and 39.4% in patients treated with sertraline plus EMM. Although patients were significantly more adherent to sertraline than other treatments, adherence rates were numerically low for all arms (sertraline with EMM 73.2%, sertraline with PE 53.6%, placebo with PE 46.3%) [58].

#### Off-label medications.

Of trials studying off-label medications for treatment of PTSD, 12 assessed clinical response [55,66,70–73,79,84–87,91], two evaluated loss of PTSD diagnosis [49,64] and three reported proportion of patients with PTSD with disease remission [66,74,87]. The rates of disease course measures are presented in Table 11.

**Table 11:** Summary of post-treatment disease course measures from off-label medications clinical trials.

Author	Year	PTSD Treatment	Rate (%)	Outcome Definition
**Clinical Response**				
Yeh [72]	2011	Topiramate	82.4%	≥30.0% reduction in CAPS score
		Placebo	64.3%	
Davis [85]	2004	Nefazodone	47.0%	
		Placebo	42.0%	
Seo [55]	2010	Paroxetine	85.0%	
		Mirtazapine	70.0%	
Monga [73]	2023	Topiramate	17.6%	CAPS score ≥20.0 points
		Placebo	5.7%	
Golier [86]	2023	Mifepristone^1^	38.1%	≥30-point reduction in total CAPS score
		Placebo^1^	31.1%	
		Mifepristone^2^	33.5%	
		Placebo^2^	39.8%	
Davidson [87]	2007	Tiagabine	49.0%^*^	CGI-C score of 1 or 2
		Placebo	54.0%^*^	
Sullivan [84]	2021	Cyclobenzaprine (5.6 mg)	63.3%	CGI-I score of 1 or 2
		Cyclobenzaprine (2.8 mg)	53.3%	
		Placebo	44.6%	
Martenyi [71]	2007	Fluoxetine 20 mg	40.5%	CGI-C score of 1 or 2 and TOP-8 criteria (at least 50.0% decrease)
		Fluoxetine 40 mg	38.8%	
		Placebo	37.5%	
Martenyi [70]	2006	Fluoxetine 80 mg	56.4%	
		Placebo	32.4%	
Abdallah [79]	2022	Ketamine 0.2 mg/kg	47.0%	≤25.0% improvement in PCL-5 at 24h post-first infusion
		Ketamine 0.5 mg/kg	47.0%	
		Placebo	33.0%	
de Kleine [66]	2012	D-cycloserine + PE ^3^	63.6%	≥10-point reduction in total CAPS score
		PE + Placebo^3^	38.2%	
		D-cycloserine + PE^4^	69.7%	
		PE + Placebo^4^	50.0%	
Davidson [91]	2003	Mirtazapine	78.6%	SPRINT global item of 1 or 2
		Placebo	14.7%	
**Loss of Diagnosis**				
van der Kolk [49]	2007	Fluoxetine	73.0%	Based on the DSM-IV criteria for PTSD
		Placebo	59.0%	
Rothbaum [64]	2014	D-cycloserine + VRE	21.4%	Based on the DSM-IV criteria for PTSD
		Placebo + VRE	26.5%	
**Remission**				
Davidson [74]	2006	Venlafaxine	50.9%	CAPS score of at least 20.0 points after treatment
		Placebo	37.5%	
Davidson [87]	2007	Tiagabine	16.0%	
		Placebo	14.0%	
de Kleine [66]	2012	D-cycloserine + PE^3^	33.3%	
		PE + Placebo^3^	26.5%	
		D-cycloserine + PE^4^	45.5%	
		PE + Placebo^4^	20.6%	

^1^ *4-week endpoint/follow-up*

^2^ *12-week endpoint*

^3^ *Post-treatment endpoint*

^4^ *3-month endpoint*

* *Difference between arms not significant*

***Abbreviations:*** *CAPS – Clinician-Administered PTSD Scale; CGI-S – Clinical Global Impressions scale – Severity; CGI-I – Clinical Global Impressions scale – Improvement; TOP-8 – Treatment Oriented PTSD Scale, 8 items; PCL-5 – PTSD Checklist for DSM-5; SPRINT – Short Posttraumatic Stress Disorder Rating Interview; DSM – Diagnostic and Statistical Manual of Mental Disorders;*

Most trials did not assess disease course outcomes (e.g., treatment response, remission rates) or explore statistical significance between study arms. Clinically meaningful improvement (i.e., treatment response) was inconsistently defined across the RCTs of off-label medications used for treatment of PTSD. There was a lack of statistical significance between interventional and control arms in almost all RCTs. Therefore, treatment response or significance was generally not reported.

Three RCTs reported PTSD remission rates, uniformly defined as a total CAPS score of at least 20.0 points after treatment. Treatment with venlafaxine was superior to placebo (50.9% vs. 37.5%, respectively, p = 0.010) and tiagabine was similar to placebo (16.0% vs. 14.0%, respectively; p = 0.880) [74,87]. D-cycloserine with PE showed a higher rate than PE control but did not assess statistical difference (33.3% vs. 26.5%, respectively; p-value not reported) [66].

#### Key summary.

Disease course outcome definitions (treatment response, remission rates, etc.) were highly heterogeneous across RCTs. Hence, results comparison of such outcomes is limited. The most uniformly defined measure was loss of PTSD diagnosis, mostly based on DSM diagnostic criteria. The percentage of patients who did not meet PTSD criteria after receiving active-dose MDMA-AT was 41.7–83.3%. The highest rate for loss of PTSD criteria was observed in 75 mg MDMA-AT controls (86.0%), while patients receiving placebo with therapy showed rates between 25.0–47.6%. Among medications, loss of diagnosis was reported only in one fluoxetine (73.0%) and one D-cycloserine with VRE (21.4%) trial. For psychotherapies, loss of diagnosis among the arms treated with varying psychotherapy modalities, rates ranged from 36.0–88.3% in CBT, 28.2–53.0% in CPT, 19.0–76.0% in EMDR, and 21.0–53.0% in PE trials. Summarized results of post-treatment disease course rates are shown in Table 12.

**Table 12:** Disease course measures after the PTSD treatments.

Author	Year	PTSD Treatment	Clinical Response (%)	Loss of PTSD diagnosis (%)	Remission (%)
**MDMA-AT**					
Mitchell [15]	2021	120-180 mg MDMA-AT^*^	90.7%	67.0%	33.0%
Mitchell [16]	2023	120-180 mg MDMA-AT^†^	86.5%	71.2%	46.2%
Mithoefer [23]	2011	125-187.5 mg MDMA-AT	83.3%	83.3%	NR
Mithoefer [24]	2018	125-187.5 mg MDMA-AT	67.0%	58.0%	NR
		75-112.5 mg MDMA-AT	100.0%	86.0%	NR
		30-45 mg MDMA-AT	29.0%	29.0%	NR
Ot’alora [25]	2018	125-187.5 mg MDMA-AT	50.0%	41.7%	NR
		100-150 mg MDMA-AT	55.6%	44.4%	NR
		40-60 mg MDMA-AT	16.7%	33.3%	NR
**Psychotherapies**					
Fecteau [33]	1999	CBT	80.0%	50.0%	NR
Beck [34]	2009	Group CBT	NR	88.3%	NR
Castillo [38]	2016	Cognitive/Exposure Therapy	NR	51.9%	NR
Ford [36]	2018	TARGET	NR	36.0%	NR
		PE	NR	21.0%	NR
Monson [32]	2012	CBCT	NR	81.0%	NR
Monson [46]	2006	CPT	47.0%	40.0%	NR
Resick [42]	2002	CPT	NR	53.0%	NR
		PE	NR	53.0%	NR
Schnurr [40]	2022	PE	NR	40.4%	NR
		CPT	NR	28.2%	NR
Ter Heide [88]	2016	EMDR	40.6%	19.0%	NR
van der Kolk [49]	2007	EMDR	NR	76.0%	NR
Taylor [48]	2003	EMDR	NR	67.0%	NR
		PE	NR	87.0%	NR
**FDA-Approved Medications**					
Marshall [52]	2001	20 mg Paroxetine	62.6%	NR	NR
		40 mg Paroxetine	56.6%	NR	NR
Seo [55]	2010	Paroxetine	85.0%	NR	NR
Li [89]	2017	Sertraline	49.0%	NR	NR
Panahi [90]	2011	Sertraline	40.0%	NR	NR
Davidson [57]	2001	Sertraline	66.5%	NR	NR
Schneier [54]	2012	Paroxetine	NR	NR	45.5%
Rauch [58]	2019	Sertraline + EMM	NR	NR	39.4%
		Sertraline + PE	NR	NR	37.7%
**Off-Label Medication**					
Yeh [72]	2011	Topiramate	82.4%	NR	NR
Monga [73]	2023	Topiramate	17.6%	NR	NR
Davis [85]	2004	Nefazodone	47.0%	NR	NR
Seo [55]	2010	Mirtazapine	70.0%	NR	NR
Golier [86]	2023	Mifepristone ^1^	33.5%	NR	NR
Davidson [87]	2007	Tiagabine	49.0%	NR	16.0%
Sullivan [84]	2021	Cyclobenzaprine (5.6 mg)	63.3%	NR	NR
		Cyclobenzaprine (2.8 mg)	53.3%	NR	NR
Martenyi [71]	2007	Fluoxetine 20 mg	40.5%	NR	NR
		Fluoxetine 40 mg	38.8%	NR	NR
Martenyi [70]	2006	Fluoxetine 80 mg	56.4%	NR	NR
Abdallah [79]	2022	Ketamine 0.2 mg/kg	47.0%	NR	NR
		Ketamine 0.5 mg/kg	47.0%	NR	NR
De Kleine [66]	2012	D-cycloserine + PE ^2^	69.7%	NR	45.5%
Davidson [91]	2003	Mirtazapine	78.6%	NR	NR
van der Kolk [49]	2007	Fluoxetine	NR	73.0%	NR
Rothbaum [64]	2014	D-Cycloserine + VRE	NR	21.4%	NR
Davidson [74]	2006	Venlafaxine	NR	NR	50.9%

* The 120–180 mg was a split dose of 80 + 40 mg for the first session and 120 + 60 mg in the second and third experimental sessions. Six participants chose either not to take the supplemental dose (n = 3, 1 MDMA) or not to escalate to the 120 mg dose (n = 3, 2 MDMA) in a total of six experimental sessions (2.3% of the total sessions across the study) [15].

† *Three participants did not undergo dose escalation in sessions 2 and 3 [16].*

***Abbreviations:*** *MDMA – 3,4-Methylenedioxymethamphetamine; MP – Manualized psychotherapy; DSM – Diagnostic and Statistical Manual Of Mental Disorders; CAPS – Clinician-Administered PTSD Scale; CBT – Cognitive Behavioral Therapy; TARGET – Trauma Affect Regulation: Guide For Education And Therapy; PE – Prolonged Exposure; CPT – Cognitive Processing Therapy; CBCT – Cognitive-Behavioral Conjoint Therapy; MA Waitlist – Minimal Attention Waitlist; VRE – Virtual Reality Exposure; EMDR – Eye Movement Desensitization and Reprocessing; NR – Not Reported*

^1^ *12-week endpoint*

^2^ *3-month endpoint*

### Treatment dropout and safety

#### MDMA-AT.

Using a standard 20.0% threshold, all reported dropout rates in MDMA-AT studies were acceptable. Phase II studies reported 7.1% [25], 9.1% [23], and 14.3% [26] dropout rates, while phase III studies showed rates of 7.8% among patients with severe PTSD [15] and 8.7% among patients with moderate or higher severity PTSD [16].

The most reported AEs in MDMA-AT trials with higher rates in the interventional arms were loss of appetite, nausea, decreased concentration, muscle tightness or weakness, and hyperhidrosis. Phase III trials did not report statistical significance in AE rates between study arms of MDMA-AT compared to placebo, and results only showed treatment-emergent adverse events (TEAEs) that occurred in >5.0% of patients treated with MDMA-AT. The most frequent AEs and also those with the greatest difference between MDMA-AT and placebo among patients with severe PTSD were muscle tightness (63.0% and 11.4%, respectively), decreased appetite (52.2% and 11.4%, respectively), nausea (30.4% and 11.4%, respectively), and hyperhidrosis (19.6% and 2.3%, respectively) [15]. The most frequent TEAEs among patients with moderate or higher severity PTSD treated with MDMA-AT were muscle tightness (58.5%), nausea (45.3%), decreased appetite (35.8%), hyperhidrosis (34.0%), and feeling hot (26.4%) [16].

During phase II and III trials, there were a few serious adverse events (SAEs) [15,23–26]. Most did not occur in the MDMA-AT arm and were not related to the study drug. The only SAE possibly related to MDMA was an acute increase in premature ventricular contractions in one patient (3.8%) during the third session of a phase II trial [24].

The impact of MDMA-AT treatment and placebo with therapy on suicidality in RCTs was assessed with the Columbia Suicide Severity Rating Scale (C-SSRS). Mithoefer et al. reported in a phase II trial among veterans, firefighters, and police officers a reduction in suicidal ideation and behavior at all endpoints (a month after the second session, two months after the third session, and at the 12-month follow-up period) [24]. A phase II trial among the general US population reported long-term benefits of three MDMA-AT doses at the 12-month endpoint, with no patients reporting serious suicidal ideation and positive suicidal behavior [25]. Following the first MDMA-AT session in a phase III study of patients with severe PTSD, positive suicidal ideation, serious suicidal ideation, and suicidal behavior rates reduced from 91.3% to 4.3%, 43.5% to 2.2%, and 34.8% to 0.0%, respectively [15]. In a confirmatory Phase III trial of patients with moderate or higher severity PTSD, positive suicidal ideation at baseline was 83.0% and 4.5% at week 18 (two participants in the MDMA-AT arm had suicidal ideation, one of whom engaged in non-suicidal self-injurious behavior) [16].

#### Psychotherapies.

Although psychotherapies were not associated with TEAEs, dropout rates were generally high in RCTs. There were only five trials with acceptable dropout rates below the 20.0% threshold in the psychotherapy arms (0.0% in CBT, 3.0% and 13.6% in CT, 13.3% in PE, and 16.7–17.2% in EMDR) [33,37,39,49,51,88]. In all other studies, treatment dropouts were 27.3–64.3% in PE [36,37,40,42,92], 20.0–46.6% in CPT [40,42,46], 21.1% in EMDR [48], 31.0% in CT [50], and 26.9–41.0% in CBT modalities [31–34,36].

#### FDA-approved medications.

There were only two paroxetine RCTs with dropout rates below the 20.0% threshold; a trial by Seo et al. [55] in Korean patients with PTSD and another by Brunet et al. [93] in a Nepali war-related PTSD population (10.0% and 13.0%, respectively). However, both studies had a small sample size (20 and 23 patients in the paroxetine arm, respectively) and shorter treatment duration compared to other trials (8 and 6 weeks, respectively) [55,93]. Other paroxetine trials (lasting between 10–22 weeks) had higher dropout rates, ranging from 32.0% to 37.9% [52,53]. Although Schneier et al. reported a 15.4% dropout rate during 12-week treatment with paroxetine, 31.6% of patients left the study during the 10-week titration period [54].

Two sertraline trials reported low dropout rates (lower than 20%), while three studies had higher than acceptable rates (between 26.1% and 30.0%) [56–58]. An Iranian study conducted by Panahi et al. [90] reported an 8.6% dropout rate in 70 war-related patients with chronic PTSD and a CGI-S score of ≥4. In an RCT by Rothbaum et al. [94], patients were highly adherent to treatment with sertraline monotherapy in combination with PE (97.1%). Dropout rates in both treatment arms were acceptable (3.2% in sertraline and 17.6% in sertraline with PE) [94]. Rauch et al. observed much greater dropout among patients treated with sertraline and PE than with sertraline only (40.6% and 26.8%, respectively) [58].

There were no SAEs reported in paroxetine and sertraline trials. All AEs were mild-to-moderate severity but inconsistently reported across the trials (S4 Table).

#### Off-label medications.

In studies with off-label PTSD medications, high treatment dropout rates (>20.0%) were reported for topiramate (55.0–58.8%), ganaxolone (54.2%), propranolol (50.0%), nefazodone (46.2%), tiagabine (33.6%), risperidone (33.3%), venlafaxine (30.4%), D-cycloserine with PE (27.3%), and low-dose cyclobenzaprine (21.1%) [66,67,73,74,76,82,84,85,87,95]. Off-label medications with RCT arms that reported acceptable dropout rates were prazosin (15.0–19.7%), divalproex (17.1%), high-dose cyclobenzaprine (16.3%), ketamine (15.0–16.0%), and risperidone (18.2%) [60,62,69,79,80,84]. Treatments with study arms reporting both lower and higher rates than an acceptable threshold (20.0%) across captured trials were D-cycloserine with VRE (0.0% and 47.2%), fluoxetine (10.0–14.5% and 21.0%), eszopiclone (14.3% and 46.2%), and mirtazapine (17.6% and 30.0%) [55,64,65,70,71,77,78,91,96].

The most common AEs related to off-label PTSD treatments were headache (4.9–60.0%), dizziness (13.0–48.0%), somnolence (5.6–33.6%), insomnia (5.0–23.0%), and diarrhea (3.9–20.0%). SAEs occurred only in ganaxolone and prazosin studies. The AE rates associated with off-label medications are presented in S4 Table.

#### Key summary.

Drop-out rates in MDMA-AT trials were acceptable, while those in psychotherapy trials were high and psychiatric medications were variable. MDMA-AT was generally well-tolerated in the PTSD population. The most frequent TEAEs in large-sample phase III trials with the greatest difference between arms were muscle tightness (63.0% and 11.4%, respectively), decreased appetite (52.2% and 11.4%, respectively), nausea (30.4% and 11.4%, respectively), and hyperhidrosis (19.6% and 2.3%, respectively). The only SAE possibly related to MDMA that occurred was an acute increase in premature ventricular contractions in one participant during the third experimental session.

A wide spectrum of AEs was noted among PTSD pharmacological interventions. FDA-approved treatment options for PTSD (paroxetine and sertraline) most frequently reported nausea (22.5–35.0%), insomnia (10.0–35.0%), headache (8.7–33.0%), and drowsiness (16.0–26.0%), while some off-label medications used for PTSD treatment commonly led to weight gain (5.0–93.3%), sedation (2.2–73.3%), and headache (4.9–60.0%). The most relevant AEs (captured in at least two studies pharmacological treatment options with >5.0% rate within intervention arms) are summarized in S4 Table. Although psychotherapies were not associated with AEs, dropout rates in related trials were mostly above the acceptable threshold (20.0%) and much higher than in other PTSD treatments.

## Discussion

This comprehensive SLR gathered and summarized the efficacy and safety evidence of available experimental treatments for chronic, treatment-resistant, moderate or higher severity PTSD. At the time of this SLR study, findings imply that MDMA-AT may be a promising innovative treatment, with desirable clinical benefits and acceptable tolerability in this population. Placebo-controlled clinical trials consistently reported a significantly greater improvement in CAPS and BDI-II scores among patients in the MDMA-AT intervention arm. The treatment was also effective in PTSD patients with borderline/diagnosed alexithymia and a low self-compassion scale score. Active-dose MDMA-AT also showed greater CAPS score decreases when compared to 25–40 mg MDMA-AT arms in all captured studies. The significant difference in BDI score changes between high- and low-dose MDMA-AT arms was inconsistently reported at post-treatment endpoints. However, 12-month follow-ups demonstrated a significant reduction in BDI scores from baseline after receiving three active-dose sessions. Additionally, a long-term trial with a mean follow-up duration of 45.4 months (range 17.0–74.0 months) reported stable clinical benefits of this experimental treatment regarding PTSD symptom severity improvement. High percentages of patients who did not meet PTSD criteria after three active-dose MDMA-AT sessions were reported in trials. As MDMA-AT was administered only up to three sessions, AEs were transient, and most were not serious or severe. The only serious AE potentially related to study treatment was premature ventricular contractions that occurred in one patient in one trial. The occurrence of serious cardiac AEs was followed in MDMA-AT clinical trials. Hence, the treatment was generally well tolerated without life-threatening or long-term consequences.

Two secondary analyses of phase II MDMA-AT trials quantitatively summarized the main results. Both studies included about 60 patients per analysis and reported a significantly higher CAPS score improvement from baseline at post-treatment endpoints after high-dose MDMA-AT (75–125 mg) than placebo or lower doses (up to 40 mg per session) [97,98]. Gorman et al. observed a 36.0-point reduction after two active dose sessions and only a 12.8-point reduction after two placebo or 30–40 mg MDMA-AT. Superiority of high-dose MDMA-AT in loss of diagnosis rates compared to placebo with manualized psychotherapy or low-dose MDMA-AT has also been shown in secondary analysis with loss of diagnosis rates of 52.3% and 33.3%, respectively [97]. Ponte et al. extended the analysis by pooling CAPS score changes after treatment cross-over and receiving the third 100–125 mg MDMA-AT session. Similar improvements in CAPS scores were reported in high-dose compared to low-dose arms (34.0 and 12.4 points decrease from baseline, respectively; p = 0.003). Two and 12 months after the final high-dose MDMA-AT session, CAPS decreased by 45.5 points and 52.1 points from baseline, respectively (both p < 0.001) [98].

FDA-approved and various off-label medications used for PTSD treatments showed highly heterogeneous findings of treatment benefits that were not statistically different from placebo administration. Only two out of six placebo-controlled RCTs of paroxetine and sertraline showed statistically significant post-treatment improvement in PTSD symptoms measured with CAPS. Likewise, most off-label medications failed to achieve statistically significance in CAPS score changes compared to placebo arms or reported inconsistent conclusions across the trials of the same medication. Propranolol, venlafaxine ER, olanzapine, nefazodone, and nabilone significantly decreased post-treatment CAPS score compared to placebo, while fluoxetine, ketamine, and cyclobenzaprine showed dose-dependent efficacy.

Although most psychotherapy trials did not assess between-group statistics, those who explored it observed CT, CPT, PE, and CBT modalities had greater post-treatment PTSD symptom improvement than waitlist controls. RCTs directly comparing multiple medications, psychotherapies, or medications and psychotherapies (paroxetine vs. mirtazapine, sertraline vs. venlafaxine ER, sertraline vs. PE, D-cycloserine with VRE vs. VRE, etc.) did not examine the superiority of one intervention over another in improving post-treatment CAPS scores. Statistically significant improvements in CAPS scores after PE compared to CPT was noted in one trial, but the difference diminished over the 6-month follow-up. Both modalities showed long-term clinical benefits (mean follow-up duration 6.2 years). BDI, SDS, and DES scores were not commonly assessed in pharmacological interventions, while disease course outcomes were inconsistently defined across trials. Higher post-treatment loss of diagnosis rates were reported in CBT modalities (CBCT and group CBT) [32–34,38]. A high percentage of patients did not meet PTSD criteria after fluoxetine treatment (73%), but a similar rate (59%) was observed in the placebo arm [49]. Pharmacological treatments were generally well tolerated, with AE rates depending on the medication, but no severe or serious events related to study interventions. Although psychotherapies are not associated with AEs, their dropout rates were much higher than in other treatments.

Previous SLRs of available PTSD treatments denoted similar findings and limitations regarding published literature evidence. Mathew et al. [99] explored the efficacy of pharmacological interventions among patients with PTSD in a quantitative summary of the results. The analysis yielded a small but statistically significant effect size of −0.23 (95% CI of −0.33 and −0.12) for selective serotonin reuptake inhibitors compared to placebo. Fluoxetine, paroxetine, and venlafaxine also demonstrated small but significant individual superiority to placebo. However, the authors pointed out a major between-study clinical and statistical heterogeneity and a small number of trials per medication which greatly limited generalizability of the findings. Heterogeneity included variances in study designs, PTSD population differences, variability of treatment characteristics, and combinations with psychotherapies [99]. SLRs with meta-analyses for psychotherapy interventions reported the strongest evidence for the efficacy of trauma-focused modalities, such as CPT, CT, and PE, inpatients with PTSD [100]. However, findings from psychotherapy studies are also limited in generalizability and internal validity by small sample sizes, lack of direct comparisons between different modalities or between psychotherapy and medications, classifying psychological interventions, high dropout rates, etc. [100–103].

FDA’s opinion on MDMA-AT for adults with PTSD was not yet determined at the time of this study. In the meantime, FDA published the Complete Response Letter requiring more clinical evidence before approval. FDA requested from Lykos Therapeutics to perform an additional phase 3 trial in order to further explore the efficacy and safety of MDMA-AT in adults with PTSD, despite the available evidence that this treatment decreased PTSD severity without serious nor severe health risks. Lykos Therapeutics announced that they will continue working towards safe and legal access to this therapy for the more than 350 million people living with PTSD worldwide [104,105].

### Strengths and limitations

This is the first, comprehensive, and up-to-date SLR that included RCTs of both psychological and pharmacological interventions in treating chronic, treatment-resistant, moderate or higher severity PTSD. Therefore, it represents a valid and relevant source of summarized efficacy and safety evidence that may be further used for other research purposes such as meta-analyses, indirect treatment comparisons, or health economic models. This study also supports clinicians, pointing out the benefits and flaws of approved and innovative treatment options for specific PTSD populations.

The main limitations are related to basic SLR design drawbacks. First, the limitations of each trial included in evidence synthesis directly influence this study’s findings. Second, although objective methods were used to minimize bias, selection, publication, and reporting biases could not be avoided for this type of research. The third limitation denotes that individual study findings might be influenced by inconsistencies due to clinical or statistical heterogeneity and imprecisions that may lead to Type I or Type II errors. Very high between-study heterogeneity was observed in population characteristics, treatment regimens, outcomes definitions, and reporting results. A substantial number of captured studies were not included in evidence synthesis as they did not report between-group statistics. Fourth, the research was designed to capture only outcomes used in MDMA-AT trials which may affected the study conclusion. Fifth, comparators of interest were chosen based on treatment guidelines. However, psychotherapies were narrowed down to the four most relevant modalities due to the wide range of available psychological interventions. Also, as the between-group statistics were used to qualitatively summarize the main findings, only studies with intervention and at least one comparator arm of interest were included. To address the risk of bias due to missing results, sources other than published reports were included in the SLR, such as public domains and clinical trial registries. Still, conference abstracts and presentations were not screened. The results of this SLR are mostly based on a small number of trials per medication with small sample sizes, without long-term follow-up assessments, and a lack of direct comparisons between relevant PTSD treatments.

## Conclusion

Three MDMA-AT sessions showed consistent clinical efficacy in reducing PTSD and depressive symptoms at post-treatment and long-term clinical endpoints in patients with chronic, treatment-resistant, moderate or higher severity PTSD. The results for frequently administered psychotherapies and pharmacological treatments (FDA-approved and off-label), provided as monotherapies or in combination, were highly heterogeneous in this population. Evidence is available for propranolol, olanzapine, venlafaxine ER, nefazodone, nabilone, BT modalities, CPT, EMDR, PE, and CT. However, the results for these medications and psychotherapies are mostly based on small sample studies without long-term follow-up assessments and with high dropout rates.

MDMA-AT and other medications used for PTSD treatment assessed in this SLR were usually followed by treatment-related AEs. However, serious AEs rarely occurred and were mostly unrelated to study medication. Additionally, MDMA-AT treatment is provided during only three manualized psychotherapy sessions with close observation and follow-up after drug administration.

Further clinical trials should be performed among a larger pool of patients with PTSD with more consistent study designs and direct head-to-head comparisons of PTSD treatment options.

## Supporting information

S1.Table SLR search strategy. (DOCX)

S2.Table NICE quality appraisal checklist results. (DOCX)

S3.Table Characteristics of studies in the SLR. (DOCX)

S4.Table Adverse events captured in ≥2 studies of pharmacological interventions for PTSD, reported in ≥5.0% of treatment arms. (DOCX)
