# Supplementary material for: MDMA-assisted therapy and current treatment options for chronic, treatment-resistant, moderate or higher severity post-traumatic stress disorder: Systematic literature review
Source: PLoS One. 2025 Jul 16;20(7):e0327778. doi: 10.1371/journal.pone.0327778 (PMC12266454; doi:10.1371/journal.pone.0327778)
Supplement: S1.Table — (DOCX) [file pone.0327778.s001.docx]

**S1 Table. SLR search strategy**

| **PubMed Search Terms** | |
| --- | --- |
| **Population** | |
| PTSD | post traumatic stress disease*[Title/Abstract] OR "post traumatic stress disorder"[Title/Abstract] OR "stress disorder post traumatic"[Title/Abstract] OR "neuroses post traumatic"[Title/Abstract] OR "Post-Traumatic Neuroses"[Title/Abstract] OR "PTSD"[Title/Abstract] OR "Posttraumatic Neuroses"[Title/Abstract] OR "post-traumatic stress disorders"[Title/Abstract] OR "post traumatic stress disorders"[Title/Abstract] OR "Posttraumatic Stress Disorders"[Title/Abstract] OR "Posttraumatic Stress Disorder"[Title/Abstract] OR "stress disorder posttraumatic"[Title/Abstract] OR "stress disorders posttraumatic"[Title/Abstract] OR "post traumatic stress disorder"[Title/Abstract] OR "stress disorder post traumatic"[Title/Abstract] OR "delayed onset post traumatic stress disorder"[Title/Abstract] OR "delayed onset posttraumatic stress disorder"[Title/Abstract] OR "chronic post traumatic stress disorder"[Title/Abstract] OR "chronic posttraumatic stress disorder"[Title/Abstract] OR "Moral Injury"[Title/Abstract] OR "injury moral"[Title/Abstract] OR "Moral Injuries"[Title/Abstract] OR "stress disorders, post traumatic"[MeSH Terms] OR "stress disorders, traumatic"[MeSH Terms] OR "stress disorder traumatic"[Title/Abstract] OR "Traumatic Stress Disorder"[Title/Abstract] OR "Traumatic Stress Disorders"[Title/Abstract] OR "war related injuries"[MeSH Terms] OR "injuries war related"[Title/Abstract] OR "injury war related"[Title/Abstract] OR "war related injuries"[Title/Abstract] OR "War-Related Injury"[Title/Abstract] OR "war related trauma"[Title/Abstract] OR "trauma war related"[Title/Abstract] OR "war related traumas"[Title/Abstract] OR "Sexual Trauma"[MeSH Terms] OR "Sexual Traumas"[Title/Abstract] OR "trauma sexual"[Title/Abstract] OR "traumas sexual"[Title/Abstract] OR "Sexual Abuse Trauma"[Title/Abstract] OR "abuse trauma sexual"[Title/Abstract] OR "Sexual Abuse Traumas"[Title/Abstract] OR "trauma sexual abuse"[Title/Abstract] OR "traumas sexual abuse"[Title/Abstract] OR "Sexual violence"[Title/Abstract] OR "trauma military sexual"[Title/Abstract] OR "Military Sexual Trauma"[MeSH Terms] OR "Combat Disorders"[MeSH Terms] OR "Combat Disorder"[Title/Abstract] OR "disorder combat"[Title/Abstract] OR "disorders combat"[Title/Abstract] OR "Combat Neurosis"[Title/Abstract] OR "Combat Neuroses"[Title/Abstract] OR "Shell Shock"[Title/Abstract] OR "shock shell"[Title/Abstract] OR "War Neurosis"[Title/Abstract] OR "War Neuroses"[Title/Abstract] OR "neuroses war"[Title/Abstract] OR "Combat Stress Disorders"[Title/Abstract] OR "Combat Stress Disorder"[Title/Abstract] OR "disorder combat stress"[Title/Abstract] OR "stress disorder combat"[Title/Abstract] OR "war induced trauma*"[Title/Abstract] OR "war induced trauma*"[Title/Abstract] OR "Post-war trauma*"[Title/Abstract] |
| **Intervention** | |
| MDMA-AT | "N-Methyl-3,4-methylenedioxyamphetamine" OR "N Methyl 3,4 methylenedioxyamphetamine" OR "MDMA" OR "Methylenedioxymethamphetamine" OR "Ecstasy Drug" OR "Ecstasy" OR "N-Methyl-3,4-methylenedioxyamphetamine"[Mesh] |
| **Comparators** | |
| FDA-approved  medications | "paroxetine" OR "Aropax" OR "BRL-29060" OR "BRL 29060" OR "BRL29060" OR "FG-7051" OR "FG 7051" OR "FG7051" OR "Paroxetine Acetate" OR "Seroxat" OR "Paroxetine Hydrochloride Anhydrous" OR "Paroxetine Maleate" OR "Paroxetine, cis-(+)-Isomer" OR "Paroxetine, cis-(-)-Isomer" OR "Paroxetine, trans-(+)-Isomer" OR "Paxil" OR "Paroxetine Hydrochloride Hemihydrate" OR "Paroxetine Hydrochloride, Hemihydrate" OR "Paroxetine Hydrochloride" OR "Paroxetine"[Mesh] OR "Sertraline"[All Fields] OR "Zoloft" OR "Lustral" OR "Altruline" OR "Apo-Sertraline" OR "Apo Sertraline" OR "Aremis" OR "Besitran" OR "Sealdin" OR "Gladem" OR "Novo-Sertraline" OR "Novo Sertraline" OR "ratio-Sertraline" OR "ratio Sertraline" OR "Rhoxal-sertraline" OR "Rhoxal sertraline" OR "Sertraline Hydrochloride" OR "Hydrochloride, Sertraline" OR "Sertraline Hydrochloride (1S-cis)-Isomer" OR "Gen-Sertraline" OR "Gen Sertraline" OR "Sertraline"[Mesh] |
| Off-label medications | "fluoxetine" OR "N-Methyl-gamma-(4-(trifluoromethyl)phenoxy)benzenepropanamine" OR "Lilly-110140" OR "Lilly 110140" OR "Sarafem" OR "Fluoxetine Hydrochloride" OR "Prozac" OR "Fluoxetine"[Mesh] OR "venlafaxine" OR "Hydrochloride, Venlafaxine" OR "Wy45030" OR "Wy-45.030" OR "Wy 45.030" OR "Sila-Venlafaxine" OR "Sila Venlafaxine" OR "Effexor" OR "Trevilor" OR "Vandral" OR "Efexor" OR "Dobupal" OR "Venlafaxine Hydrochloride"[Mesh] OR "Escitalopram" OR "Escitalopram Oxalate" OR "Lexapro" OR "Escitalopram"[Mesh] OR "Nefazodone" OR "Serzone" OR "Dutonin" OR "Nefadar" OR "nefazodone hydrochloride" OR "Lin-Nefazodone" OR "Menfazona" OR "Rulivan" OR "Apo-Nefazodone" OR "nefazodone" [Supplementary Concept] OR "imipramine" OR "Norchlorimipramine" OR "Imidobenzyle" OR "Imizin" OR "Tofranil" OR "Janimine" OR "Melipramine" OR "Pryleugan" OR "Imipramine Pamoate" OR "4,4'-Methylenebis(3-hydroxy-2-naphthoic acid)-3-(10,11-dihydro-5H-dibenzo(b,f)azepin-5-yl)-N,N-dimethyl-1-propanamine (1:2)" OR "Imipramine Hydrochloride" OR "Imipramine Monohydrochloride" OR "Imipramine"[Mesh] OR "amitriptyline" OR "Tryptine" OR "Amineurin" OR "Amitrip" OR "Amitriptylin Beta" OR "Amitriptylin Desitin" OR "Desitin, Amitriptylin" OR "Amitriptylin RPh" OR "RPh, Amitriptylin" OR "Amitriptylin-Neuraxpharm" OR "Amitriptylin Neuraxpharm" OR "Amitriptyline Hydrochloride" OR "Amitrol" OR "Anapsique" OR "Apo-Amitriptyline" OR "Apo Amitriptyline" OR "Damilen" OR "Domical" OR "Laroxyl" OR "Lentizol" OR "Novoprotect" OR "Saroten" OR "Sarotex" OR "Syneudon" OR "Triptafen" OR "Endep" OR "Tryptizol" OR "Elavil" OR "Tryptanol" OR "Amitriptyline"[Mesh] OR "mirtazapine" OR "6-Azamianserin" OR "6 Azamianserin" OR "ORG 3770" OR "ORG-3770" OR "ORG3770" OR "Rexer" OR "Remeron" OR "Norset" OR "Remergil" OR "Zispin" OR "Esmirtazapine" OR "(S)-Mirtazapine" OR "Org 50081" OR "(N-Methyl-11C)mirtazapine" OR "Mirtazapine"[Mesh] OR "phenelzine" OR "beta-Phenylethylhydrazine" OR "beta Phenylethylhydrazine" OR "Fenelzin" OR "Phenethylhydrazine" OR "2-Phenethylhydrazine" OR "2 Phenethylhydrazine" OR "Phenelzine Sulfate" OR "Sulfate, Phenelzine" OR "Nardelzine" OR "Nardil" OR "Phenelzine"[Mesh] OR "Brofaromine" OR "4-(5-methoxy-7-bromobenzofuranyl)-2-piperidine" OR "brofaromine hydrochloride" OR "CGP 11305A" OR "CGP-11305A" OR "brofaromine" [Supplementary Concept] OR "Risperidone" OR "Risperdal Consta" OR "Consta, Risperidal" OR "Risperidal" OR "R-64,766" OR "R 64,766" OR "R64,766" OR "R-64766" OR "R 64766" OR "R64766" OR "Risperidone"[Mesh] OR "Quetiapine" OR "Quetiapine Fumarate" OR "Ethanol, 2-(2-(4-dibenzo(b,f)(1,4)thiazepin-11-yl-1-piperazinyl)ethoxy)-, (E)-2-butenedioate (2:1) (salt)" OR "Seroquel" OR "ICI 204,636" OR "ICI-204636" OR "ICI204636" OR "ICI 204636" OR "2-(2-(4-dibenzo(b,f)(1,4)thiazepine-11-yl-1-piperazinyl)ethoxy)ethanol" OR "Quetiapine Fumarate"[Mesh] OR "olanzapine" OR "2-Methyl-4-(4-methyl-1-piperazinyl)-10H-thieno(2,3-b)(1,5)benzodiazepine" OR "LY-170052" OR "LY 170052" OR "LY170052" OR "Zyprexa" OR "Zolafren" OR "LY 170053" OR "Olanzapine Pamoate" OR "Olanzapine"[Mesh] OR "topiramate" OR "2,3-4,5-bis-O-(1-methylethylidene)-beta-D-fructopyranose sulfamate" OR "Epitomax" OR "McN 4853" OR "McN-4853" OR "McN4853" OR "USL255" OR "Topamax" OR "Topiramate"[Mesh] OR "lamotrigine" OR "3,5-Diamino-6-(2,3-dichlorophenyl)-as-triazine" OR "3,5-Diamino-6-(2,3-dichlorophenyl)-1,2,4-triazine" OR "Lamictal" OR "Crisomet" OR "Lamiktal" OR "BW-430C" OR "BW 430C" OR "BW430C" OR "Labileno" OR "Lamotrigine"[Mesh] OR "tiagabine" OR "N-(4,4-di(3-Methylthien-2-yl)but-3-enyl)nipecotic acid" OR "Gabitril" OR "NO 329" OR "NO-329" OR "Tiagabine Hydrochloride" OR "(R)-(4,4-bis(3-Methyl-2-thienyl)-3-butenyl)-3-piperidinecarboxylic acid, Hydrochloride" OR "Tiagabine, (S)-isomer" OR "NO 328" OR "NO-328" OR "Tiagabine"[Mesh] OR "ganaxolone" OR "3alpha-hydroxy-3beta-methyl-5alpha-pregnan-20-one" OR "CCD 1042" OR "CCD-1042" OR "Ztalmy" OR "ganaxolone" [Supplementary Concept] OR "divalproex" OR "2-Propylpentanoic Acid" OR "2 Propylpentanoic Acid" OR "Depakene" OR "Convulsofin" OR "Depakote" OR "Dipropyl Acetate" OR "Divalproex Sodium" OR "Semisodium Valproate" OR "Vupral" OR "Propylisopropylacetic Acid" OR "Ergenyl" OR "Magnesium Valproate" OR "Valproate" OR "Valproate Sodium" OR "Sodium Valproate" OR "Calcium Valproate" OR "Valproate Calcium" OR "Depakine" OR "Valproic Acid"[Mesh] OR "ketamine" OR "2-(2-Chlorophenyl)-2-(methylamino)cyclohexanone" OR "CI-581" OR "CI 581" OR "CI581" OR "Ketalar" OR "Ketaset" OR "Ketanest" OR "Calipsol" OR "Kalipsol" OR "Calypsol" OR "Ketamine Hydrochloride" OR "Ketamine"[Mesh] OR "prazosin" OR "Furazosin" OR "Prazosin Hydrochloride" OR "Hydrochloride, Prazosin" OR "Prazosin HCL" OR "HCL, Prazosin" OR "Minipress" OR "Pratsiol" OR "Prazosin"[Mesh] OR "Propranolol" OR "Inderal" OR "Avlocardyl" OR "AY-20694" OR "AY 20694" OR "Dexpropranolol" OR "Dociton" OR "Obsidan" OR "Obzidan" OR "Propranolol Hydrochloride" OR "Hydrochloride, Propranolol" OR "Anaprilin" OR "Anapriline" OR "Betadren" OR "Propranolol"[Mesh] OR "mifepristone" OR "ZK-98296" OR "ZK 98296" OR "ZK98296" OR "R-38486" OR "R 38486" OR "RU-486" OR "RU 486" OR "RU486" OR "R38486" OR "RU-38486" OR "RU 38486" OR "RU38486" OR "Mifegyne" OR "Mifégyne" OR "Mifeprex" OR "Mifepristone"[Mesh] OR "D-cycloserine" OR "R-4-Amino-3-isoxazolidinone" OR "Seromycin" OR "Cycloserine"[Mesh] OR "cyclobenzaprine" OR "Flexeril" OR "Lisseril" OR "cyclobenzaprine hydrochloride" OR "cyclobenzaprine" [Supplementary Concept] OR "Cannabidiol" OR "1,3-Benzenediol, 2-(3-methyl-6-(1-methylethenyl)-2-cyclohexen-1-yl)-5-pentyl-, (1R-trans)-" OR "Epidiolex" OR "Cannabidiol"[Mesh] OR "Dronabinol" OR "delta(9)-THC" OR "9-ene-Tetrahydrocannabinol" OR "9 ene Tetrahydrocannabinol" OR "THC" OR "delta(1)-Tetrahydrocannabinol" OR "delta(1)-THC" OR "delta(9)-Tetrahydrocannabinol" OR "Tetrahydrocannabinol" OR "Tetrahydrocannabinol, (6a-trans)-Isomer" OR "Tetrahydrocannabinol, Trans-Isomer" OR "Tetrahydrocannabinol, Trans Isomer" OR "Tetrahydrocannabinol, (6aS-cis)-Isomer" OR "Tetrahydrocannabinol, Trans-(+-)-Isomer" OR "Marinol" OR "Tetrahydrocannabinol, (6aR-cis)-Isomer" OR "Dronabinol"[Mesh] OR ((((((("Bupropion" OR "Bupropion"[Mesh] OR "Amfebutamone" OR "Wellbutrin" OR "Zyban" OR "Zyntabac") OR ("Buspirone" OR "Buspirone"[Mesh] OR "MJ-9022" OR "MJ 9022" OR "Busp" OR "Buspar" OR "Bespar")) OR ("Citalopram" OR "Citalopram"[Mesh] OR "Lu-10-171" OR "Lu10171" OR "Seropram" OR "Celexa")) OR ("Desvenlafaxine" OR "Desvenlafaxine Succinate"[Mesh] OR "desmethylvenlafaxine" OR "Pristiq" OR "4-(2-(dimethylamino)-1-(1-hydroxycyclohexyl)ethyl)phenol")) OR (((("eszopiclone") OR ("Lunesta")) OR ("Estorra")) OR ("Eszopiclone"[Mesh]))) OR (((((((((((("pregabalin") OR ("(S)-3-(aminomethyl)-5-methylhexanoic acid")) OR ("3-isobutyl GABA")) OR ("3 isobutyl GABA")) OR ("3-(aminomethyl)-5-methylhexanoic acid")) OR ("(R-)-3-isobutyl GABA")) OR ("(S+)-3-isobutyl GABA")) OR ("Lyrica")) OR ("CI 1008")) OR ("CI-1008")) OR ("CI1008")) OR ("Pregabalin"[Mesh]))) OR ((((((((("rivastigmine") OR ("Rivastigmine Hydrogen Tartrate")) OR ("Exelon")) OR ("ENA 713")) OR ("SDZ ENA 713")) OR ("ENA 713, SDZ")) OR ("ENA-713")) OR ("ENA713")) OR ("Rivastigmine"[Mesh]))) OR ((((((((((("Duloxetine") OR ("Hydrochloride, Duloxetine")) OR ("Duloxetine HCl")) OR ("HCl, Duloxetine")) OR ("LY 248686")) OR ("LY-248686")) OR ("LY248686")) OR ("LY227942")) OR ("N-methyl-3-(1-naphthalenyloxy)-2-thiophenepropanamine")) OR ("Cymbalta")) OR ("Duloxetine Hydrochloride"[Mesh])) |
| Psychotherapies | ((("cognitive behavior therap*"[Title/Abstract] OR "cognitive therap*"[Title/Abstract] OR "behavior therap*"[Title/Abstract] OR "cognitive behavioral therap*"[Title/Abstract] OR "behavioral therap*"[Title/Abstract] OR "cognitive behaviour therap*"[Title/Abstract] OR "behaviour therap*"[Title/Abstract] OR "cognitive behavioural therap*"[Title/Abstract] OR "behavioural therap*"[Title/Abstract] OR "cognitive behavior therap*"[Title/Abstract] OR "cognitive behaviour therap*"[Title/Abstract] OR "cognition therap*"[Title/Abstract] OR "cognitive therap*"[Title/Abstract] OR "cognitive behavior psychotherap*"[Title/Abstract] OR "cognitive psychotherap*"[Title/Abstract] OR "behavior psychotherap*"[Title/Abstract] OR "cognitive behavioral psychotherap*"[Title/Abstract] OR "behavioral psychotherap*"[Title/Abstract] OR "behaviour psychotherap*"[Title/Abstract] OR "cognitive behavioural psychotherap*"[Title/Abstract] OR "behavioural psychotherap*"[Title/Abstract] OR "cognitive behavior psychotherap*"[Title/Abstract] OR "cognitive behavioral therapy"[MeSH Terms] OR "cognitive behavior counsel*"[Title/Abstract] OR "cognitive counsel*"[Title/Abstract] OR "behavior counsel*"[Title/Abstract] OR "cognitive behavioral counsel*"[Title/Abstract] OR "behavioral counsel*"[Title/Abstract] OR "behaviour counsel*"[Title/Abstract] OR "cognitive behavioural counsel*"[Title/Abstract] OR "behavioural counsel*"[Title/Abstract] OR "cognitive behavior counsel*"[Title/Abstract] OR "cognitive counsel*"[Title/Abstract]) OR ("cognitive processing therap*"[Title/Abstract] OR "cognitive processing approach*"[Title/Abstract])) OR ("prolonged exposure"[Title/Abstract])) OR ("Trauma-focused therap*"[Title/Abstract] OR "trauma-focused treatment*"[Title/Abstract] OR "trauma-focused counsel*"[Title/Abstract] OR "trauma-focused intervention*"[Title/Abstract] OR "trauma-focused approach*"[Title/Abstract] OR "trauma-focused psychotherap*"[Title/Abstract] OR "Trauma-oriented therap*"[Title/Abstract] OR "Trauma-informed therap*"[Title/Abstract] OR "trauma-informed treatment*"[Title/Abstract] OR "trauma-informed counsel*"[Title/Abstract] OR "trauma-informed intervention*"[Title/Abstract] OR "trauma-informed approach*"[Title/Abstract] OR "trauma-informed psychotherap*"[Title/Abstract]) OR ("Eye Movement Desensitization and Processing"[All Fields] OR "EMDR"[All Fields] OR "Eye Movement Desensitization Reprocessing"[MeSH Terms]) |
| **Outcomes** | |
| Outcomes | (((((((((("Clinician Administered PTSD Scale"[Title/Abstract] OR "CAPS-5"[Title/Abstract] OR "CAPS-IV"[Title/Abstract] OR "Clinician Administered posttraumatic stress disorder Scale"[Title/Abstract] OR "CAPS-V"[Title/Abstract] OR "CAPS-4"[Title/Abstract]) OR ("Beck Depression"[Title/Abstract] OR "BDI-II"[Title/Abstract] OR "Beck's Depression"[Title/Abstract])) OR ("Dissociative Experience scale"[Title/Abstract] OR "dissociation experience scale"[Title/Abstract] OR "Dissociative Experiences scale"[Title/Abstract] OR "DES-II"[Title/Abstract])) OR ("Sheehan Disability Scale"[Title/Abstract] OR "Sheehan Disability Scales"[Title/Abstract])) OR ("Columbia Suicide Severity Rating Scale"[Title/Abstract] OR "C-SSRS"[Title/Abstract] OR "Columbia Suicide Severity Rating Scales"[Title/Abstract])) OR ("survival rate*"[Title/Abstract] OR "survival time*"[Title/Abstract] OR "year survival"[Title/Abstract] OR "years survival"[Title/Abstract] OR "month survival"[Title/Abstract] OR "months survival"[Title/Abstract] OR "median survival"[Title/Abstract] OR "mean survival"[Title/Abstract] OR "Survival rate"[MeSH Terms])) OR ("Mortality"[Title/Abstract] OR "Mortalities"[Title/Abstract] OR "fatality rate*"[Title/Abstract] OR "death*"[Title/Abstract] OR "fatal outcome*"[Title/Abstract] OR "Mortality"[MeSH Terms])) OR ("adverse event*"[Title/Abstract] OR "adverse reaction*"[Title/Abstract] OR "adverse effect*"[Title/Abstract] OR "side effect*"[Title/Abstract] OR "toxicit*"[Title/Abstract] OR "Drug-Related Side Effects and Adverse Reactions"[MeSH Terms])) OR ("suicide*"[Title/Abstract] OR "Suicidal"[Title/Abstract] OR "parasuicide*"[Title/Abstract] OR "Parasuicidal"[Title/Abstract] OR "fatal attempt*"[Title/Abstract] OR "self harm*"[Title/Abstract] OR "self destructi*"[Title/Abstract] OR "self injur*"[Title/Abstract] OR "Suicide"[MeSH Terms] OR "Suicidal ideation"[MeSH Terms] OR "suicide, attempted"[MeSH Terms] OR "suicide, completed"[MeSH Terms])) OR ("Relapse"[Title/Abstract] OR "relapsing"[Title/Abstract] OR "relapses"[Title/Abstract] OR "Recurrence"[Title/Abstract] OR "reoccurrence"[Title/Abstract] OR "Recrudescence"[Title/Abstract] OR "Recurrence"[MeSH Terms] OR "progression"[Title/Abstract] OR "exacerbation"[Title/Abstract] OR "Disease Progression"[MeSH Terms] OR "deterioration"[Title/Abstract] OR "remission"[Title/Abstract] OR "recovery"[Title/Abstract] OR "regression"[Title/Abstract] OR "patient dropouts"[MeSH Terms] OR "dropout"[Title/Abstract] OR "dropouts"[Title/Abstract])) OR ("Adherence"[Title/Abstract] OR "Adherent"[Title/Abstract] OR "Proportion of days covered"[Title/Abstract] OR "PDC"[Title/Abstract] OR "medication possession ratio*"[Title/Abstract] OR "MPR"[Title/Abstract] OR "Medication Adherence"[MeSH Terms] OR "Treatment Adherence and Compliance"[MeSH Terms] OR "Persistence"[Title/Abstract] OR "Persistent"[Title/Abstract] OR "prescription gap*"[Title/Abstract] OR "treatment gap*"[Title/Abstract] OR "therapy gap*"[Title/Abstract] OR "gap analys*"[Title/Abstract] OR "Augmentation"[Title/Abstract] OR "concomitant medication*"[Title/Abstract] OR "concomitant drug*"[Title/Abstract] OR "concomitant therap*"[Title/Abstract] OR "concomitant treatment*"[Title/Abstract] OR "Concomitant use"[Title/Abstract] OR "Compliance"[Title/Abstract] OR "Compliant"[Title/Abstract] OR "Patient Compliance"[MeSH Terms] OR "treatment pattern*"[Title/Abstract] OR "therapy pattern*"[Title/Abstract] OR "treatment duration*"[Title/Abstract] OR "therapy duration*"[Title/Abstract] OR "treatment discontinuation*"[Title/Abstract] OR "therapy discontinuation*"[Title/Abstract] OR "treatment switch*"[Title/Abstract] OR "therapy switch*"[Title/Abstract] OR "treatment transition*"[Title/Abstract] OR "therapy transition*"[Title/Abstract] OR "treatment failure*"[Title/Abstract] OR "therapy failure*"[Title/Abstract] OR "treatment interruption*"[Title/Abstract] OR "therapy interruption*"[Title/Abstract] OR "dose increas*"[Title/Abstract] OR "dose escalation*"[Title/Abstract] OR "dose reduction*"[Title/Abstract] OR "dose interruption*"[Title/Abstract]) |
| **Embase Search Terms** | |
| **Population** | |
| PTSD | 'posttraumatic stress disorder'/exp OR 'posttraumatic stress disorder':ti,kw,ab OR 'combat stress'/exp OR 'combat stress':ti,kw,ab OR 'shell shock'/exp OR 'shell shock':ti,kw,ab OR 'moral injury'/exp OR 'moral injury':ti,kw,ab OR 'sexual trauma'/exp OR 'sexual trauma':ti,kw,ab OR 'sexual violence'/exp OR 'sexual violence':ti,kw,ab OR 'child abuse'/exp OR 'child abuse':ti,kw,ab OR 'psychotrauma'/exp OR 'psychotrauma':ti,kw,ab OR 'psychotraum*':ti,kw,ab OR 'battle trauma'/exp OR 'battle trauma':ti,kw,ab OR 'war trauma'/exp OR 'war trauma':ti,kw,ab OR 'war-related trauma'/exp OR 'war-related trauma':ti,kw,ab |
| **Intervention** | |
| MDMA-AT | 'midomafetamine'/exp OR 'ecstasy'/exp OR 'ecstasy' OR 'ecstacy'/exp OR 'ecstacy' OR 'mdma' OR 'methylenedioxymethamphetamine' OR 'mdma-at' OR 'mdma-assisted therap*' OR 'mdma-assisted psychotherap*' |
| **Comparators** | |
| FDA-approved  medications | 'sertraline'/exp OR 'sertraline' OR 'paroxetine'/exp OR 'paroxetine' |
| Off-label medications | 'fluoxetine'/exp OR 'fluoxetine' OR 'venlafaxine'/exp OR 'venlafaxine' OR 'escitalopram'/exp OR 'escitalopram' OR 'nefazodone'/exp OR 'nefazodone' OR 'imipramine'/exp OR 'imipramine' OR 'amitriptyline'/exp OR 'amitriptyline' OR 'mirtazapine'/exp OR 'mirtazapine' OR 'phenelzine'/exp OR 'phenelzine' OR 'brofaromine'/exp OR 'brofaromine' OR 'risperidone'/exp OR 'risperidone' OR 'quetiapine'/exp OR 'quetiapine' OR 'olanzapine'/exp OR 'olanzapine' OR 'topiramate'/exp OR 'topiramate' OR 'lamotrigine'/exp OR 'lamotrigine' OR 'tiagabine'/exp OR 'tiagabine' OR 'ganaxolone'/exp OR 'ganaxolone' OR 'valproic acid'/exp OR 'valproic acid' OR 'valproate semisodium'/exp OR 'valproate semisodium' OR 'valproate magnesium'/exp OR 'valproate magnesium' OR 'valproate calcium'/exp OR 'valproate calcium' OR 'ketamine'/exp OR 'ketamine' OR 'ketamin'/exp OR 'ketamin' OR 'prazosin'/exp OR 'prazosin' OR 'propranolol'/exp OR 'propranolol' OR 'dexpropranolol'/exp OR 'dexpropranolol' OR 'mifepristone'/exp OR 'mifepristone' OR 'cycloserine'/exp OR 'cycloserine' OR 'cyclobenzaprine'/exp OR 'cyclobenzaprine' OR 'cannabidiol'/exp OR 'cannabidiol' OR 'cannabidiolic acid'/exp OR 'cannabidiolic acid' OR 'dronabinol'/exp OR 'dronabinol' OR '11 hydroxydronabinol'/exp OR '11 hydroxydronabinol' OR 'nabiximols'/exp OR 'nabiximols' OR 'amfebutamone'/exp OR 'amfebutamone' OR 'buspirone'/exp OR 'buspirone' OR 'citalopram'/exp OR 'citalopram' OR 'desvenlafaxine'/exp OR 'desvenlafaxine' OR 'eszopiclone'/exp OR 'eszopiclone' OR 'pregabalin'/exp OR 'pregabalin' OR 'rivastigmine'/exp OR 'rivastigmine' OR 'duloxetine'/exp OR 'duloxetine' OR 'duloxetine oxalate'/exp OR 'duloxetine oxalate' |
| Psychotherapies | 'cognitive behavioral therapy'/exp OR 'cognitive behavioral therapy' OR 'cognitive behavioral stress management'/exp OR 'cognitive behavioral stress management' OR 'cognitive behavioral conjoint therapy'/exp OR 'cognitive behavioral conjoint therapy' OR 'cognitive behavioral group therapy'/exp OR 'cognitive behavioral group therapy' OR 'behavio$ral therap*' OR 'behavio$ral treatment*' OR 'behavio$ral counsel*' OR 'behavio$ral intervention*' OR 'behavio$ral approach*' OR 'behavio$ral psychotherap*' OR 'cognitive processing therapy'/exp OR 'cognitive processing therapy' OR 'cognitive processing therap*' OR 'cognitive processing treatment*' OR 'cognitive processing counsel*' OR 'cognitive processing intervention*' OR 'cognitive processing approach*' OR 'cognitive processing psychotherap*' OR 'prolonged exposure therapy'/exp OR 'prolonged exposure therapy' OR 'prolonged exposure'/exp OR 'prolonged exposure' OR 'trauma-focused cognitive behavioral therapy'/exp OR 'trauma-focused cognitive behavioral therapy' OR 'trauma-focused therap*' OR 'trauma-focused treatment*' OR 'trauma-focused counsel*' OR 'trauma-focused intervention*' OR 'trauma-focused approach*' OR 'trauma-focused psychotherap*' OR 'trauma-oriented therap*' OR 'trauma-informed therap*' OR 'trauma-informed treatment*' OR 'trauma-informed counsel*' OR 'trauma-informed intervention*' OR 'trauma-informed approach*' OR 'trauma-informed psychotherap*' OR 'eye movement desensitization and reprocessing'/exp OR 'eye movement desensitization and reprocessing' OR 'emdr' |
| **Outcomes** | |
| Outcomes | 'clinician administered ptsd':ti,kw,ab OR 'clinician administered post traumatic stress':ti,kw,ab OR 'clinician administered posttraumatic stress':ti,kw,ab OR 'caps-4':ti,kw,ab OR 'caps4':ti,kw,ab OR 'caps-iv':ti,kw,ab OR 'caps-5':ti,kw,ab OR 'caps5':ti,kw,ab OR 'caps-v':ti,kw,ab OR 'beck depression inventory'/exp OR 'beck depression inventory':ti,kw,ab OR 'becker$ depression':ti,kw,ab OR 'beck$ depression':ti,kw,ab OR 'bdi-ii':ti,kw,ab OR 'bdi-2':ti,kw,ab OR 'bdi2':ti,kw,ab OR 'dissociative experiences scale'/exp OR 'dissociative experiences scale':ti,kw,ab OR 'dissociative experience$ scale':ti,kw,ab OR 'dissociation experience$ scale':ti,kw,ab OR 'des-ii':ti,kw,ab OR 'sheehan disability scale'/exp OR 'sheehan disability scale':ti,kw,ab OR 'sheehan$ disability':ti,kw,ab OR 'columbia suicide severity rating scale'/exp OR 'columbia suicide severity rating scale':ti,kw,ab OR 'columbia suicide severity rating':ti,kw,ab OR 'c-ssrs':ti,kw,ab OR 'adverse event'/exp OR 'adverse event':ti,kw,ab OR 'adverse event$':ti,kw,ab OR 'adverse effect$':ti,kw,ab OR 'adverse reaction$':ti,kw,ab OR 'adverse drug reaction'/exp OR 'adverse drug reaction':ti,kw,ab OR 'adverse drug event$':ti,kw,ab OR 'adverse drug effect$':ti,kw,ab OR 'adverse drug reaction$':ti,kw,ab OR 'side effect'/exp OR 'side effect':ti,kw,ab OR 'side effect$':ti,kw,ab OR 'side reaction$':ti,kw,ab OR 'toxicity'/exp OR 'toxicity':ti,kw,ab OR 'toxicit*':ti,kw,ab OR 'suicidal behavior'/exp OR 'suicidal behavior':ti,kw,ab OR 'suicid*':ti,kw,ab OR 'parasuicid*':ti,kw,ab OR 'fatal attempt*':ti,kw,ab OR 'self harm*':ti,kw,ab OR 'self destructi*':ti,kw,ab OR 'self injur*':ti,kw,ab OR 'mortality'/exp OR 'mortality':ti,kw,ab OR 'mortalit*':ti,kw,ab OR 'death'/exp OR 'death':ti,kw,ab OR 'death*':ti,kw,ab OR 'fatal* rate$':ti,kw,ab OR 'fatal* outcome$':ti,kw,ab OR 'survival'/exp OR 'survival':ti,kw,ab OR 'relapse'/exp OR 'relapse':ti,kw,ab OR 'relaps*':ti,kw,ab OR 'recurrent disease'/exp OR 'recurrent disease':ti,kw,ab OR 'recurrence risk'/exp OR 'recurrence risk':ti,kw,ab OR 'recurrence$':ti,kw,ab OR 'recurrent':ti,kw,ab OR 'reoccurrence$':ti,kw,ab OR 'recrudescence'/exp OR 'recrudescence':ti,kw,ab OR 'recrudescen*':ti,kw,ab OR 'progression'/exp OR 'progression':ti,kw,ab OR 'exacerbation'/exp OR 'exacerbation':ti,kw,ab OR 'disease exacerbation'/exp OR 'disease exacerbation':ti,kw,ab OR 'deterioration'/exp OR 'deterioration':ti,kw,ab OR 'remission'/exp OR 'remission':ti,kw,ab OR 'recovery'/exp OR 'recovery':ti,kw,ab OR 'disease regression'/exp OR 'disease regression':ti,kw,ab OR 'dropouts'/exp OR 'dropouts':ti,kw,ab OR 'patient dropout'/exp OR 'patient dropout':ti,kw,ab OR 'dropout*':ti,kw,ab OR 'dropping out':ti,kw,ab OR 'patient compliance'/exp OR 'patient compliance':ti,kw,ab OR 'adherence'/exp OR 'adherence':ti,kw,ab OR 'adherence to refills and medications scale'/exp OR 'adherence to refills and medications scale':ti,kw,ab OR 'adherent':ti,kw,ab OR 'proportion of days covered'/exp OR 'proportion of days covered':ti,kw,ab OR 'proportion$ of days cover*':ti,kw,ab OR 'medication possession ratio'/exp OR 'medication possession ratio':ti,kw,ab OR 'medication$ possession ratio$':ti,kw,ab OR 'persistence'/exp OR 'persistence':ti,kw,ab OR 'persisten*':ti,kw,ab OR 'prescription$ gap*':ti,kw,ab OR 'treatment$ gap*':ti,kw,ab OR 'therap* gap*':ti,kw,ab OR 'gap analys*':ti,kw,ab OR 'augmentation'/exp OR 'augmentation':ti,kw,ab OR 'augmentation therapy'/exp OR 'augmentation therapy':ti,kw,ab OR 'concomitant therapy'/exp OR 'concomitant therapy':ti,kw,ab OR 'concomitant therap*':ti,kw,ab OR 'concomitant treatment$':ti,kw,ab OR 'concomitant medication$':ti,kw,ab OR 'concomitant drug$':ti,kw,ab OR 'concomitant use$':ti,kw,ab OR 'compliance$':ti,kw,ab OR 'compliant':ti,kw,ab OR 'treatment pattern$':ti,kw,ab OR 'treatment duration'/exp OR 'treatment duration':ti,kw,ab OR 'treatment duration*':ti,kw,ab OR 'treatment discontinuation*':ti,kw,ab OR 'treatment switching'/exp OR 'treatment switching':ti,kw,ab OR 'treatment switch*':ti,kw,ab OR 'treatment transition*':ti,kw,ab OR 'treatment failure'/exp OR 'treatment failure':ti,kw,ab OR 'treatment fail*':ti,kw,ab OR 'treatment interruption'/exp OR 'treatment interruption':ti,kw,ab OR 'treatment interrupt*':ti,kw,ab OR 'therap* pattern$':ti,kw,ab OR 'therap* duration*':ti,kw,ab OR 'therap* discontinuation*':ti,kw,ab OR 'therap* switch*':ti,kw,ab OR 'therap* transition*':ti,kw,ab OR 'therap* fail*':ti,kw,ab OR 'therap* interrupt*':ti,kw,ab OR 'drug dose increase'/exp OR 'drug dose increase':ti,kw,ab OR 'dos* increas*':ti,kw,ab OR 'drug dose escalation'/exp OR 'drug dose escalation':ti,kw,ab OR 'dos* escalat*':ti,kw,ab OR 'drug dose reduction'/exp OR 'drug dose reduction':ti,kw,ab OR 'dos* reduct*':ti,kw,ab OR 'drug dose intensification'/exp OR 'drug dose intensification':ti,kw,ab OR 'dos* intensi*':ti,kw,ab OR 'dose densification'/exp OR 'dose densification':ti,kw,ab OR 'dos* densit*':ti,kw,ab |
