# Supplementary material for: MDMA-assisted therapy and current treatment options for chronic, treatment-resistant, moderate or higher severity post-traumatic stress disorder: Systematic literature review
Source: PLoS One. 2025 Jul 16;20(7):e0327778. doi: 10.1371/journal.pone.0327778 (PMC12266454; doi:10.1371/journal.pone.0327778)
Supplement: S2.Table — (DOCX) [file pone.0327778.s002.docx]

**Supplementary Table S2. NICE quality appraisal checklist results**

| **Study Identification (First Author, and Publication Year)** | **Abdallah, 2022** | **Akbarian, 2015** | **Bartzokis, 2005** | **Brunet, 2014** | **Brunet, 2018** | **Brunet, 2021** | **Bryant, 2018** | **Buhmann, 2018** | **Carey, 2012** | **Castillo, 2016** |
| --- | --- | --- | --- | --- | --- | --- | --- | --- | --- | --- |
| **Section 1: Population** |  |  |  |  |  |  |  |  |  |  |
| **1.1 Is the source population or source area well described?** | ++ | + | ++ | + | ++ | ++ | ++ | ++ | ++ | ++ |
| **1.2 Is the eligible population or area representative of the source population or area?** | ++ | + | + | + | + | + | + | + | + | + |
| **1.3 Do the selected participants or areas represent the eligible population or area?** | ++ | ++ | ++ | NR | ++ | ++ | ++ | ++ | ++ | + |
| **Section 2: Method of allocation to intervention (or comparison)** |  |  |  |  |  |  |  |  |  |  |
| **2.1 Allocation to intervention (or comparison). How was selection bias minimized?** | ++ | ++ | ++ | ++ | ++ | ++ | ++ | ++ | ++ | ++ |
| **2.2 Were interventions (and comparisons) well described and appropriate?** | ++ | ++ | ++ | ++ | ++ | ++ | ++ | ++ | ++ | ++ |
| **2.3 Was the allocation concealed?** | ++ | ++ | ++ | ++ | ++ | ++ | ++ | ++ | ++ | ++ |
| **2.4 Were participants or investigators blind to exposure and comparison?** | ++ | + | ++ | NR | ++ | ++ | + | + | ++ | + |
| **2.5 Was the exposure to the intervention and comparison adequate?** | ++ | ++ | ++ | ++ | ++ | ++ | ++ | ++ | ++ | ++ |
| **2.6 Was contamination acceptably low?** | ++ | ++ | ++ | ++ | ++ | ++ | ++ | ++ | ++ | ++ |
| **2.7 Were other interventions similar in both groups?** | ++ | ++ | ++ | ++ | ++ | ++ | ++ | + | ++ | ++ |
| **2.8 Were all participants accounted for at the study conclusion?** | ++ | ++ | + | NR | + | ++ | ++ | + | ++ | ++ |
| **2.9 Did the setting reflect usual UK practice?** | ++ | ++ | ++ | NA | ++ | ++ | ++ | ++ | ++ | ++ |
| **2.10 Did the intervention or control comparison reflect usual UK practice?** | ++ | ++ | ++ | NA | ++ | ++ | ++ | ++ | ++ | ++ |
| **Section 3: Outcomes** |  |  |  |  |  |  |  |  |  |  |
| **3.1 Were outcome measures reliable?** | ++ | ++ | ++ | ++ | ++ | ++ | ++ | ++ | ++ | ++ |
| **3.2 Were all outcome measurements complete?** | ++ | ++ | ++ | ++ | ++ | ++ | ++ | ++ | ++ | ++ |
| **3.3 Were all important outcomes assessed?** | ++ | + | + | + | + | + | + | + | ++ | ++ |
| **3.4 Were outcomes relevant?** | ++ | + | ++ | ++ | ++ | ++ | ++ | ++ | ++ | ++ |
| **3.5 Were there similar follow-up times in exposure and comparison groups?** | ++ | ++ | ++ | ++ | ++ | ++ | ++ | ++ | ++ | ++ |
| **3.6 Was follow-up time meaningful?** | + | + | + | + | + | + | ++ | ++ | + | ++ |
| **Section 4: Analyses** |  |  |  |  |  |  |  |  |  |  |
| **4.1 Were exposure and comparison groups similar at baseline? If not, were these adjusted?** | ++ | ++ | ++ | NR | ++ | + | NR | + | ++ | + |
| **4.2 Was the intention to treat (ITT) analysis conducted?** | ++ | ++ | ++ | NR | + | ++ | ++ | ++ | ++ | ++ |
| **4.3 Was the study sufficiently powered to detect an intervention effect (if one exists)?** | ++ | ++ | ++ | NR | NR | ++ | ++ | ++ | ++ | ++ |
| **4.4 Were the estimates of effect size given or calculable?** | ++ | ++ | NR | NR | ++ | ++ | ++ | ++ | ++ | ++ |
| **4.5 Were the analytical methods appropriate?** | ++ | ++ | ++ | ++ | ++ | ++ | ++ | ++ | ++ | ++ |
| **4.6 Was the precision of intervention effects given or calculable? Were they meaningful?** | ++ | ++ | ++ | ++ | ++ | + | ++ | ++ | ++ | ++ |
| **Section 5: Summary** |  |  |  |  |  |  |  |  |  |  |
| **5.1 Are the study results internally valid (i.e., unbiased)?** | ++ | ++ | ++ | + | ++ | ++ | ++ | ++ | ++ | ++ |
| **5.2 Are the findings generalizable to the source population (i.e., externally valid)?** | ++ | + | + | + | + | + | + | + | + | + |

**Supplementary Table S2. NICE quality appraisal checklist results (continue)**

| **Davidson, 2003** | **Davidson, 2006** | **Davidson, 2007** | **Davis, 2004** | **Davis, 2008** | **Difede, 2014** | **Foa, 2005** | **Forbes, 2012** | **Ford, 2018** | **Gorman, 2020** | **Gutner, 2016** | **Jetly, 2015** | **Krystal, 2012** | **Li, 2017** |
| --- | --- | --- | --- | --- | --- | --- | --- | --- | --- | --- | --- | --- | --- |
|  |  |  |  |  |  |  |  |  |  |  |  |  |  |
| ++ | ++ | ++ | ++ | ++ | ++ | ++ | ++ | ++ | ++ | ++ | ++ | ++ | ++ |
| + | ++ | ++ | + | + | + | ++ | + | + | + | ++ | + | ++ | + |
| ++ | ++ | ++ | ++ | ++ | ++ | ++ | ++ | ++ | ++ | ++ | ++ | ++ | ++ |
|  |  |  |  |  |  |  |  |  |  |  |  |  |  |
| ++ | ++ | ++ | ++ | ++ | ++ | ++ | ++ | ++ | ++ | ++ | ++ | ++ | ++ |
| ++ | ++ | ++ | ++ | ++ | ++ | ++ | ++ | ++ | NA | ++ | ++ | ++ | ++ |
| ++ | ++ | ++ | ++ | ++ | ++ | ++ | ++ | ++ | NA | ++ | ++ | ++ | ++ |
| + | ++ | ++ | ++ | ++ | ++ | + | + | + | NA | + | ++ | ++ | ++ |
| ++ | ++ | ++ | ++ | ++ | ++ | ++ | ++ | ++ | ++ | ++ | ++ | ++ | ++ |
| ++ | ++ | ++ | ++ | ++ | ++ | ++ | ++ | ++ | ++ | ++ | ++ | ++ | ++ |
| ++ | ++ | ++ | ++ | ++ | ++ | ++ | ++ | ++ | ++ | ++ | ++ | ++ | ++ |
| + | + | ++ | + | ++ | ++ | + | ++ | + | ++ | ++ | ++ | ++ | ++ |
| ++ | NA | ++ | ++ | ++ | ++ | ++ | ++ | ++ | ++ | ++ | ++ | ++ | ++ |
| ++ | NA | ++ | ++ | ++ | ++ | ++ | ++ | ++ | ++ | ++ | ++ | ++ | ++ |
|  |  |  |  |  |  |  |  |  |  |  |  |  |  |
| ++ | ++ | ++ | ++ | ++ | ++ | ++ | ++ | ++ | ++ | ++ | ++ | ++ | ++ |
| ++ | ++ | + | ++ | ++ | ++ | ++ | ++ | ++ | ++ | ++ | ++ | ++ | ++ |
| + | ++ | + | + | + | + | + | + | + | + | + | + | + | + |
| ++ | ++ | ++ | ++ | ++ | ++ | ++ | ++ | ++ | ++ | ++ | ++ | ++ | ++ |
| ++ | ++ | ++ | ++ | ++ | ++ | ++ | ++ | ++ | NA | ++ | ++ | ++ | ++ |
| + | ++ | + | + | + | ++ | ++ | ++ | + | + | ++ | + | ++ | + |
|  |  |  |  |  |  |  |  |  |  |  |  |  |  |
| ++ | ++ | ++ | ++ | ++ | ++ | + | ++ | ++ | ++ | ++ | ++ | ++ | ++ |
| ++ | ++ | ++ | ++ | ++ | ++ | ++ | ++ | ++ | ++ | ++ | ++ | ++ | ++ |
| ++ | ++ | ++ | ++ | ++ | ++ | ++ | ++ | ++ | NA | ++ | ++ | ++ | ++ |
| ++ | ++ | ++ | ++ | ++ | ++ | ++ | ++ | ++ | NA | ++ | ++ | ++ | ++ |
| ++ | ++ | ++ | ++ | ++ | ++ | ++ | ++ | ++ | NA | ++ | ++ | ++ | ++ |
| ++ | ++ | ++ | ++ | ++ | ++ | ++ | ++ | ++ | ++ | ++ | ++ | ++ | ++ |
|  |  |  |  |  |  |  |  |  |  |  |  |  |  |
| ++ | ++ | ++ | ++ | ++ | ++ | ++ | ++ | ++ | + | ++ | ++ | ++ | ++ |
| + | ++ | ++ | + | + | + | ++ | + | + | + | ++ | + | ++ | + |

**Supplementary Table S2. NICE quality appraisal checklist results (continue)**

| **Lindley, 2007** | **Lloyd, 2014** | **Marshall, 2007** | **McDonagh, 2005** | **Mitchell, 2021** | **Mithoefer, 2011** | **Mithoefer, 2013** | **Mithoefer, 2018** | **Monson, 2006** | **Monson, 2012** | **Nacasch, 2011** | **Oehen, 2013** | **Ot'alora, 2018** | **Padala, 2006** | **Panahi, 2011** | **Ponte, 2021** |
| --- | --- | --- | --- | --- | --- | --- | --- | --- | --- | --- | --- | --- | --- | --- | --- |
|  |  |  |  |  |  |  |  |  |  |  |  |  |  |  |  |
| ++ | ++ | ++ | ++ | ++ | ++ | ++ | ++ | ++ | ++ | ++ | ++ | ++ | + | ++ | ++ |
| + | + | + | + | + | + | + | + | + | + | + | + | + | + | + | + |
| ++ | ++ | ++ | ++ | ++ | ++ | ++ | ++ | ++ | ++ | ++ | ++ | ++ | ++ | ++ | ++ |
|  |  |  |  |  |  |  |  |  |  |  |  |  |  |  |  |
| ++ | ++ | ++ | ++ | ++ | ++ | ++ | ++ | ++ | ++ | ++ | ++ | ++ | ++ | ++ | ++ |
| ++ | ++ | ++ | ++ | ++ | ++ | ++ | ++ | ++ | ++ | ++ | ++ | ++ | ++ | ++ | ++ |
| ++ | ++ | ++ | ++ | ++ | ++ | ++ | ++ | NR | ++ | ++ | ++ | ++ | ++ | ++ | ++ |
| ++ | + | ++ | + | ++ | ++ | ++ | ++ | + | + | + | ++ | ++ | ++ | ++ | ++ |
| ++ | ++ | ++ | ++ | ++ | ++ | ++ | ++ | ++ | ++ | ++ | ++ | ++ | ++ | ++ | ++ |
| ++ | ++ | ++ | ++ | ++ | ++ | ++ | ++ | ++ | ++ | ++ | ++ | ++ | ++ | ++ | ++ |
| ++ | ++ | ++ | ++ | ++ | ++ | ++ | ++ | ++ | ++ | ++ | ++ | ++ | ++ | ++ | ++ |
| + | ++ | + | + | ++ | ++ | ++ | ++ | ++ | + | ++ | ++ | ++ | + | ++ | ++ |
| ++ | ++ | ++ | ++ | ++ | ++ | ++ | ++ | ++ | ++ | ++ | ++ | ++ | ++ | ++ | ++ |
| ++ | ++ | ++ | ++ | ++ | ++ | ++ | ++ | ++ | ++ | ++ | ++ | ++ | ++ | ++ | ++ |
|  |  |  |  |  |  |  |  |  |  |  |  |  |  |  |  |
| ++ | ++ | ++ | ++ | ++ | ++ | ++ | ++ | ++ | ++ | ++ | ++ | ++ | ++ | ++ | ++ |
| ++ | ++ | ++ | ++ | ++ | ++ | ++ | ++ | ++ | ++ | ++ | ++ | ++ | ++ | ++ | ++ |
| + | + | + | + | ++ | ++ | ++ | ++ | ++ | ++ | + | + | ++ | + | + | + |
| ++ | ++ | ++ | ++ | ++ | ++ | ++ | ++ | ++ | ++ | ++ | ++ | ++ | ++ | ++ | ++ |
| ++ | ++ | ++ | ++ | ++ | ++ | ++ | ++ | ++ | ++ | ++ | ++ | ++ | ++ | ++ | ++ |
| + | ++ | + | ++ | ++ | + | ++ | ++ | + | + | ++ | + | ++ | + | + | ++ |
|  |  |  |  |  |  |  |  |  |  |  |  |  |  |  |  |
| ++ | NR | NR | + | ++ | + | + | + | ++ | + | ++ | ++ | + | + | + | + |
| ++ | ++ | ++ | ++ | ++ | ++ | ++ | ++ | ++ | ++ | ++ | ++ | ++ | ++ | ++ | ++ |
| ++ | ++ | ++ | ++ | ++ | ++ | ++ | + | ++ | ++ | NR | + | ++ | NR | NR | NA |
| ++ | ++ | ++ | ++ | ++ | ++ | ++ | ++ | ++ | ++ | ++ | ++ | ++ | - | ++ | ++ |
| ++ | ++ | ++ | ++ | ++ | ++ | ++ | ++ | ++ | ++ | ++ | ++ | ++ | ++ | ++ | ++ |
| ++ | + | ++ | ++ | ++ | ++ | ++ | ++ | ++ | ++ | ++ | ++ | ++ | + | ++ | ++ |
|  |  |  |  |  |  |  |  |  |  |  |  |  |  |  |  |
| ++ | + | ++ | ++ | ++ | ++ | ++ | ++ | ++ | ++ | ++ | ++ | ++ | + | ++ | ++ |
| + | + | + | + | + | ++ | ++ | ++ | ++ | ++ | ++ | ++ | ++ | + | ++ | ++ |

**Supplementary Table S2. NICE quality appraisal checklist results (continue)**

| **Raskind, 2003** | **Raskind, 2007** | **Raskind, 2013** | **Raskind, 2018** | **Rasmusson, 2017** | **Rauch, 2019** | **Resick, 2002** | **Resick, 2012** | **Rothbaum, 2006** | **Rothbaum, 2014** | **Schneier, 2012** | **Schnurr, 2022** | **Seo, 2010** | **Sullivan, 2021** | **Van der Kolk, 2023** | **Van der Kolk, 2007** | **Yeh, 2011** | **Zohar, 2002** |
| --- | --- | --- | --- | --- | --- | --- | --- | --- | --- | --- | --- | --- | --- | --- | --- | --- | --- |
|  |  |  |  |  |  |  |  |  |  |  |  |  |  |  |  |  |  |
| + | ++ | ++ | ++ | ++ | ++ | ++ | ++ | ++ | ++ | ++ | ++ | ++ | ++ | ++ | ++ | ++ | + |
| + | + | + | ++ | ++ | ++ | ++ | ++ | + | ++ | + | ++ | + | ++ | ++ | + | + | + |
| ++ | ++ | ++ | ++ | ++ | ++ | ++ | ++ | ++ | ++ | ++ | ++ | ++ | ++ | ++ | ++ | ++ | ++ |
|  |  |  |  |  |  |  |  |  |  |  |  |  |  |  |  |  |  |
| ++ | ++ | ++ | ++ | ++ | ++ | ++ | ++ | ++ | ++ | ++ | ++ | ++ | ++ | ++ | ++ | ++ | ++ |
| ++ | ++ | ++ | ++ | ++ | ++ | ++ | ++ | ++ | ++ | ++ | ++ | ++ | ++ | ++ | ++ | ++ | ++ |
| ++ | ++ | ++ | ++ | ++ | ++ | ++ | ++ | ++ | ++ | ++ | ++ | ++ | ++ | ++ | ++ | ++ | ++ |
| ++ | ++ | ++ | ++ | ++ | ++ | + | + | + | ++ | ++ | ++ | + | ++ | ++ | ++ | ++ | ++ |
| ++ | ++ | ++ | ++ | ++ | ++ | ++ | ++ | ++ | ++ | ++ | ++ | ++ | ++ | ++ | ++ | ++ | ++ |
| ++ | ++ | ++ | ++ | ++ | ++ | ++ | ++ | ++ | ++ | ++ | ++ | ++ | ++ | ++ | ++ | ++ | ++ |
| ++ | ++ | ++ | ++ | ++ | ++ | ++ | ++ | ++ | ++ | ++ | ++ | ++ | ++ | ++ | ++ | ++ | ++ |
| NR | ++ | + | ++ | + | + | + | + | + | + | + | + | + | + | ++ | ++ | + | + |
| ++ | ++ | ++ | ++ | ++ | ++ | ++ | ++ | ++ | ++ | ++ | ++ | ++ | ++ | ++ | ++ | ++ | ++ |
| ++ | ++ | ++ | ++ | ++ | ++ | ++ | ++ | ++ | ++ | ++ | ++ | ++ | ++ | ++ | ++ | ++ | ++ |
|  |  |  |  |  |  |  |  |  |  |  |  |  |  |  |  |  |  |
| ++ | ++ | ++ | ++ | ++ | ++ | ++ | ++ | ++ | ++ | ++ | ++ | ++ | ++ | ++ | ++ | ++ | ++ |
| ++ | ++ | ++ | ++ | ++ | ++ | ++ | ++ | ++ | ++ | ++ | ++ | ++ | ++ | ++ | ++ | ++ | ++ |
| + | + | + | + | + | ++ | + | ++ | + | + | ++ | + | ++ | ++ | ++ | + | ++ | ++ |
| ++ | ++ | ++ | ++ | ++ | ++ | ++ | ++ | ++ | ++ | ++ | ++ | ++ | ++ | ++ | ++ | ++ | ++ |
| ++ | ++ | ++ | ++ | ++ | ++ | ++ | ++ | ++ | ++ | ++ | ++ | ++ | ++ | ++ | ++ | ++ | ++ |
| + | + | + | + | + | + | ++ | ++ | + | ++ | ++ | ++ | + | + | ++ | ++ | + | + |
|  |  |  |  |  |  |  |  |  |  |  |  |  |  |  |  |  |  |
| NR | NR | ++ | ++ | + | + | ++ | NR | NR | ++ | + | ++ | ++ | ++ | ++ | ++ | ++ | + |
| ++ | ++ | ++ | ++ | ++ | ++ | ++ | ++ | ++ | ++ | ++ | ++ | ++ | ++ | ++ | ++ | ++ | ++ |
| NR | NR | NR | ++ | ++ | ++ | ++ | ++ | NR | NR | NR | ++ | ++ | + | ++ | NR | NR | NR |
| ++ | ++ | ++ | ++ | ++ | ++ | ++ | ++ | ++ | ++ | ++ | ++ | ++ | ++ | ++ | ++ | ++ | ++ |
| ++ | ++ | ++ | ++ | ++ | ++ | ++ | ++ | ++ | ++ | ++ | ++ | ++ | ++ | ++ | ++ | ++ | ++ |
| ++ | ++ | ++ | ++ | ++ | ++ | ++ | ++ | ++ | ++ | ++ | ++ | ++ | ++ | ++ | ++ | ++ | ++ |
|  |  |  |  |  |  |  |  |  |  |  |  |  |  |  |  |  |  |
| + | ++ | ++ | ++ | ++ | ++ | ++ | ++ | ++ | ++ | ++ | ++ | ++ | ++ | ++ | ++ | ++ | + |
| ++ | ++ | ++ | ++ | ++ | ++ | ++ | ++ | ++ | ++ | ++ | ++ | ++ | ++ | ++ | ++ | ++ | ++ |

**Supplementary Table S2. NICE quality appraisal checklist results (continue)**

| **Duran, 2020** | **Ehlers, 2005** | | | **Fecteau, 1999** | | **Marshall, 2001** | **Martenyi, 2006** | **Martenyi, 2007** | **Davidson, 2001** | **Barnett, 2002** | **Beck, 2009** | | | **Bryant, 2011** | | **Davidson, 2006** | **de Kleine, 2012** | | | **Duffy, 2007** |  |
| --- | --- | --- | --- | --- | --- | --- | --- | --- | --- | --- | --- | --- | --- | --- | --- | --- | --- | --- | --- | --- | --- |
|  | |  |  | |  | |  |  |  |  | |  |  | |  | | |  |  | |  |
| + | | ++ | + | | ++ | | ++ | ++ | ++ | ++ | | + | ++ | | ++ | | | ++ | ++ | |  |
| + | | + | + | | ++ | | ++ | ++ | ++ | + | | + | + | | ++ | | | + | + | |  |
| ++ | | ++ | ++ | | ++ | | ++ | ++ | ++ | ++ | | + | ++ | | ++ | | | ++ | + | |  |
|  | |  |  | |  | |  |  |  |  | |  |  | |  | | |  |  | |  |
| ++ | | ++ | ++ | | ++ | | ++ | ++ | ++ | ++ | | ++ | ++ | | ++ | | | ++ | ++ | |  |
| ++ | | ++ | ++ | | ++ | | ++ | ++ | ++ | ++ | | ++ | ++ | | ++ | | | ++ | ++ | |  |
| ++ | | ++ | ++ | | ++ | | ++ | ++ | ++ | ++ | | ++ | ++ | | ++ | | | ++ | ++ | |  |
| ++ | | ++ | ++ | | ++ | | ++ | ++ | ++ | ++ | | ++ | ++ | | ++ | | | ++ | ++ | |  |
| ++ | | ++ | ++ | | ++ | | ++ | ++ | ++ | ++ | | ++ | ++ | | ++ | | | ++ | ++ | |  |
| ++ | | ++ | ++ | | ++ | | ++ | ++ | ++ | ++ | | ++ | ++ | | ++ | | | ++ | ++ | |  |
| ++ | | ++ | ++ | | ++ | | ++ | ++ | ++ | ++ | | ++ | ++ | | ++ | | | ++ | ++ | |  |
| + | | ++ | ++ | | + | | ++ | ++ | + | + | | + | ++ | | ++ | | | + | + | |  |
| ++ | | ++ | ++ | | ++ | | ++ | ++ | ++ | ++ | | ++ | ++ | | ++ | | | ++ | ++ | |  |
| ++ | | ++ | ++ | | ++ | | ++ | ++ | ++ | ++ | | ++ | ++ | | ++ | | | ++ | ++ | |  |
|  | |  |  | |  | |  |  |  |  | |  |  | |  | | |  |  | |  |
| ++ | | ++ | ++ | | ++ | | ++ | ++ | ++ | ++ | | ++ | ++ | | ++ | | | ++ | ++ | |  |
| ++ | | ++ | ++ | | ++ | | ++ | ++ | ++ | ++ | | ++ | ++ | | ++ | | | ++ | ++ | |  |
| ++ | | ++ | ++ | | + | | ++ | ++ | ++ | + | | ++ | ++ | | ++ | | | + | ++ | |  |
| ++ | | ++ | ++ | | ++ | | ++ | ++ | ++ | ++ | | ++ | ++ | | ++ | | | ++ | ++ | |  |
| ++ | | ++ | ++ | | ++ | | ++ | ++ | ++ | ++ | | ++ | ++ | | ++ | | | ++ | ++ | |  |
| ++ | | ++ | ++ | | + | | ++ | + | + | + | | ++ | + | | + | | | ++ | ++ | |  |
|  | |  |  | |  | |  |  |  |  | |  |  | |  | | |  |  | |  |
| ++ | | ++ | NR | | ++ | | ++ | ++ | ++ | ++ | | ++ | ++ | | ++ | | | ++ | ++ | |  |
| + | | ++ | ++ | | ++ | | ++ | ++ | ++ | ++ | | ++ | ++ | | ++ | | | ++ | ++ | |  |
| ++ | | ++ | NR | | NR | | ++ | ++ | ++ | ++ | | NR | + | | ++ | | | ++ | ++ | |  |
| ++ | | ++ | ++ | | ++ | | ++ | ++ | ++ | ++ | | ++ | ++ | | ++ | | | ++ | ++ | |  |
| ++ | | ++ | NR | | ++ | | ++ | ++ | ++ | ++ | | ++ | ++ | | ++ | | | ++ | ++ | |  |
| ++ | | ++ | ++ | | ++ | | ++ | ++ | ++ | ++ | | ++ | ++ | | ++ | | | ++ | ++ | |  |
|  | |  |  | |  | |  |  |  |  | |  |  | |  | | |  |  | |  |
| + | | + | + | | + | | ++ | ++ | ++ | ++ | | + | ++ | | ++ | | | ++ | + | |  |
| ++ | | ++ | + | | ++ | | ++ | ++ | ++ | + | | ++ | ++ | | ++ | | | + | ++ | |  |

**Supplementary Table S2. NICE quality appraisal checklist results (continue)**

| **Acarturk, 2015** | **Dowd, 2020** | **Golier, 2023** | **Mitchell, 2023** | **Monga, 2023** | **Pollack, 2011** | **Taylor, 2003** | **Ter Heide, 2016** |
| --- | --- | --- | --- | --- | --- | --- | --- |
|  |  |  |  |  |  |  |  |
| ++ | ++ | ++ | ++ | + | + | + | ++ |
|  |  |  |  | Only demographics | Brief demographics and clinical. | Brief demographics and clinical. |  |
| + | + | + | ++ | + | + | + | + |
| Small sample size (n=29) | Small sample size (n=25) | Small sample size (n=85) |  | Small sample size (n=68). | Small sample size (n=24). | Small sample size (n=60). | Small sample size (n=72). |
|  |  |  |  |  |  |  |  |
| + | + | ++ | ++ | ++ | ++ | ++ | ++ |
| Syrian refugees |  |  |  |  |  |  |  |
|  |  |  |  |  |  |  |  |
| ++ | ++ | ++ | ++ | ++ | ++ | ++ | ++ |
| ++ | ++ | ++ | ++ | ++ | ++ | ++ | ++ |
| ++ | ++ | ++ | ++ | ++ | ++ | ++ | ++ |
| ++ | ++ | ++ | ++ | ++ | ++ | ++ | ++ |
| ++ | ++ | ++ | ++ | ++ | ++ | ++ | ++ |
| ++ | ++ | ++ | ++ | ++ | ++ | ++ | ++ |
| ++ | ++ | ++ | ++ | ++ | ++ | ++ | ++ |
| ++ | + | ++ | ++ | + | ++ | + | + |
| ++ | ++ | ++ | ++ | ++ | ++ | ++ | ++ |
| ++ | ++ | ++ | ++ | ++ | ++ | ++ | ++ |
|  |  |  |  |  |  |  |  |
| ++ | ++ | ++ | ++ | ++ | ++ | ++ | ++ |
| ++ | ++ | ++ | ++ | ++ | ++ | ++ | ++ |
| + | ++ | ++ | ++ | ++ | ++ | ++ | ++ |
| ++ | ++ | ++ | ++ | ++ | ++ | ++ | ++ |
| ++ | ++ | ++ | ++ | ++ | ++ | ++ | ++ |
| ++ | ++ | ++ | ++ | ++ | + | ++ | ++ |
|  |  |  |  |  |  |  |  |
| ++ | ++ | ++ | ++ | ++ | ++ | ++ | ++ |
| ++ | ++ | ++ | ++ | ++ | ++ | ++ | ++ |
| ++ | ++ | ++ | ++ | + | NR | NR | ++ |
| ++ | ++ | ++ | ++ | ++ | ++ | ++ | ++ |
| ++ | ++ | ++ | ++ | ++ | ++ | ++ | ++ |
| ++ | ++ | ++ | ++ | ++ | ++ | ++ | ++ |
|  |  |  |  |  |  |  |  |
| ++ | ++ | ++ | ++ | + | + | + | ++ |
| + | + | + | ++ | ++ | + | + | ++ |
