# Supplementary material for: MDMA-assisted therapy and current treatment options for chronic, treatment-resistant, moderate or higher severity post-traumatic stress disorder: Systematic literature review
Source: PLoS One. 2025 Jul 16;20(7):e0327778. doi: 10.1371/journal.pone.0327778 (PMC12266454; doi:10.1371/journal.pone.0327778)
Supplement: S3.Table — (DOCX) [file pone.0327778.s003.docx]

**S3 Table.** **Characteristics of studies in the SLR**

| **Authors** | **Country** | **Publication Year** | **Subpopulation Type** | **Population Size** | **Baseline Severity Evaluation Tool** | **Intervention** | **Comparator** |
| --- | --- | --- | --- | --- | --- | --- | --- |
| Abdallah [79] | US | 2022 | Veterans and active members | 158 patients | CAPS-5 | Ketamine | Placebo |
| Acarturk [47] | Turkey | 2015 | Syrian refugees | 29 patients | IES-R | EMDR | Waitlist |
| Akbarian [29] | Iran | 2015 | - | 28 patients | IES-R | CBT | MC waitlist |
| Barnett [96] | US | 2002 | - | 65 patients | SCID & SIP | Fluoxetine | Placebo |
| Bartzokis [67] | US | 2005 | Veterans | 65 patients | CAPS | Risperidone | Placebo |
| Beck [34] | US | 2009 | MVA survivors | 44 patients | CAPS | Group CBT | Minimum contact comparison |
| Brunet [75] | NR | 2014 | - | 41 patients | CAPS | Propranolol + TMR | Placebo + TMR |
| Brunet [76] | Canada | 2018 | - | 60 patients | PCL-S | Propranolol + TMR | Placebo + TMR |
| Brunet [93] | Nepal | 2021 | Dang district patients | 46 patients | PCL | Paroxetine | Propranolol + RT |
| Bryant [30] | Australia | 2019 | Emergency service personnel | 100 patients | CAPS | CBT | Waitlist |
| Bryant [35] | Thailand | 2011 | Terrorist-related PTSD | 28 patients | PSS-I | CBT | TAU |
| Buhmann [104] | Denmark | 2018 | Refugees with war-related PTSD and no psychoses | 280 patients | HTQ | Sertraline | 1. CBT 2. Sertraline + CBT 3. Waiting list |
| Carey [83] | South Africa | 2012 | Non-combat PTSD | 28 patients | CAPS | Olanzapine | Placebo |
| Castillo [38] | US | 2016 | Female veterans | 86 patients | CAPS | Group CET | Waitlist |
| Davidson [91] | US | 2003 | - | 26 patients | SIP | Mirtazapine | Placebo |
| Davidson [74] | Global | 2006 | - | 329 patients | CAPS-SX | Venlafaxine ER | Placebo |
| Davidson [87] | US | 2007 | - | 227 patients | CAPS and DTS | Tiagabine | Placebo |
| Davidson [57] | US | 2001 | Outpatient civilians | 202 patients | CAPS-2 | Sertraline | Placebo |
| Davidson [59] | US | 2006 | - | 531 patients | CAPS-SX and DTS | Venlafaxine ER | 1. Sertraline 2. Placebo |
| Davis [85] | US | 2004 | Veterans and 1 civilian | 42 patients | CAPS | Nefazodone | Placebo |
| Davis [80] | US | 2008 | Veterans | 82 patients | CAPS | Divalproex sodium | Placebo |
| Dowd [78] | US | 2020 | with sleep disturbances | 25 patients | CAPS | Eszopiclone | Placebo |
| de Kleine [66] | Netherlands | 2012 | - | 67 patients | CAPS-1 | D-cycloserine + PE | Placebo + PE |
| Difede [65] | US | 2014 | WTC survivors | 25 patients | CAPS | D-Cycloserine + VRE | Placebo + VRE |
| Duffy [50] | Northern Ireland | 2007 | - | 58 patients | PDS | CT | Waitlist |
| Duran [37] | Brazil | 2020 | - | 95 patients | DTS | PE | CBT |
| Ehlers [51] | UK | 2005 | - | 28 patients | CAPS | CT | Waitlist |
| Fecteau [33] | - | 1999 | MVA survivors | 20 patients | CAPS-2 | CBT | Waitlist |
| Foa [92] | US | 2005 | Women | 179 patients | PSS-I | PE | 1. PE + CR 2. Waitlist |
| Forbes [44] | Australia | 2012 | Veterans | 59 patients | CAPS | CPT | TAU |
| Ford [36] | US | 2018 | Male veterans with anger issues | 31 patients | CAPS | TARGET | PE |
| Golier [86] | US | 2023 | Male veterans | 80 patients | CAPS | Mifepristone | Placebo |
| Gutner [41] | US | 2016 | Women | 136 patients | CAPS | CPT | PE |
| Jetly [81] | Canada | 2015 | Male military personnel with trauma-related nightmares | 19 patients | CAPS | Nabilone | Placebo |
| Krystal [68] | US | 2011 | Veterans | 267 patients | CAPS | Risperidone | Placebo |
| Li [89] | China | 2017 | - | 72 patients | IES-R | Sertraline | Placebo |
| Lindley [95] | US | 2007 | Vietnam veterans | 40 patients | CAPS | Topiramate | Placebo |
| Lloyd [45] | Australia | 2014 | Veterans | 59 patients | CAPS | CPT | TAU |
| Marshall [53] | US | 2007 | Urban population | 52 patients | CAPS-2 | Paroxetine | Placebo |
| Marshall [52] | US | 2001 | - | 551 patients | CAPS-2 | Paroxetine (20 and 40 mg) | Placebo |
| Martenyi [70] | Ex-Yugoslavia | 2006 | Veterans | 144 patients | CAPS-2 | Fluoxetine | Placebo |
| Martenyi [71] | US | 2007 | - | 411 patients | CAPS | Fluoxetine (20 mg and 40 mg) | Placebo |
| McDonagh [31] | - | 2005 | Women with childhood sexual abuse | 74 patients | CAPS | CBT | 1. PCT 2. Waitlist |
| Mitchell [15] | US, Canada, Israel | 2021 | Severe PTSD | 90 patients | CAPS-5 | MDMA -AT | Placebo + MP |
| Mitchell [16] | US and Israel | 2023 | Moderate to severe PTSD | 104 patients | CAPS-5 | MDMA-AT | Placebo + MP |
| Mithoefer [23] | US | 2011 | - | 20 patients | CAPS-IV | MDMA-AT | Placebo + MP |
| Mithoefer [27] | US | 2013 | - | 16 Phase II pilot study completers | - | MDMA-AT | - |
| Mithoefer [24] | US | 2018 | Veterans, firefighters, or police officers | 26 patients | CAPS-IV | MDMA-AT (125 mg) | MDMA-AT (30 mg and 75 mg) |
| Monga [73] | US | 2023 | Civilian patients with PTSD | 72 patients | CAPS | Topiramate | Placebo |
| Monson [46] | US | 2006 | Veterans | 60 patients | CAPS | CPT | Waitlist |
| Monson [32] | US & Canada | 2012 | Couples with one PTSD partner | 80 patients | CAPS | CBCT | Waitlist |
| Nacasch [39] | Israel | 2011 | Veterans | 30 patients | PSS-I | PE | TAU |
| Oehen [26] | Switzerland | 2013 | - | 12 patients | CAPS | MDMA-AT (125 mg) | MDMA-AT (25 mg) |
| Ot'alora [25] | US | 2018 | - | 28 patients | CAPS-IV | MDMA-AT (100 mg and 125 mg) | MDMA-AT (40 mg) |
| Padala [69] | US | 2006 | Women | 20 patients | CAPS | Risperidone | Placebo |
| Panahi [90] | Iran | 2011 | Veterans | 70 patients | IES-R | Sertraline | Placebo |
| Pollack [77] | US | 2011 | with insomnia | 27 patients | CAPS | Eszopiclone | Placebo |
| Raskind [61] | US | 2003 | Vietnam veterans | 10 patients | CAPS | Prazosin | Placebo |
| Raskind [62] | US | 2007 | Veterans | 40 patients | CAPS | Prazosin | Placebo |
| Raskind [63] | US | 2013 | Active-duty members & recently discharged veterans | 67 patients | CAPS | Prazosin | Placebo |
| Raskind [60] | US | 2018 | Veterans | 304 patients | CAPS | Prazosin | Placebo |
| Rasmusson [82] | US | 2017 | - | 112 patients | CAPS | Ganaxolone | Placebo |
| Rauch [58] | US | 2019 | Veterans and active service members | 207 patients | CAPS | Sertraline | 1. Sertraline + PE 2. Placebo + PE |
| Resick [42] | US | 2002 | Female rape victims | 171 patients | CAPS | CPT | 1. PE 2. Minimal attention waitlist |
| Resick [43] | US | 2012 | Female rape victims | 126 parent study completers | - | CPT | PE |
| Rothbaum [94] | US | 2006 | - | 88 patients | SIP | Sertraline + PE | Sertraline |
| Rothbaum [64] | US | 2014 | Veterans (22-55 years old) | 156 patients | CAPS | 1. D-Cycloserine + VRE | 1. Placebo + VRE 2. Alprazolam + VRE |
| Schneier [54] | US | 2012 | WTC survivors | 37 patients | CAPS | Paroxetine + PE | Placebo + PE |
| Schnurr [40] | US | 2022 | Veterans | 916 patients | CAPS-5 | PE | CPT |
| Seo [55] | Korea | 2010 | - | 40 patients | CAPS-2 | Mirtazapine | Paroxetine |
| Sullivan [84] | US | 2021 | Military -related PTSD | 231 patients | CAPS-5 | Cyclobenzaprine (2.8 mg and 5.6mg) | Placebo |
| Taylor [48] | Canada | 2003 | - | 60 patients | CAPS | EMDR |  |
| Ter Heide [88] | The Netherlands | 2016 | Refugees | 72 patients | CAPS | EMDR | TAU |
| van der Kolk [28] | US, Canada, Israel | 2023 | - | 90 patients | CAPS-5 | MDMA-AT | Placebo + MP |
| van der Kolk [49] | US | 2007 | - | 88 patients | CAPS | Fluoxetine | 1. Placebo 2. EMDR |
| Yeh [72] | Brazil | 2011 | Civilians | 35 patients | CAPS | Topiramate | Placebo |
| Zohar [56] | Israel | 2002 | Israeli veterans | 42 patients | CAPS-2 | Sertraline | Placebo |

**Abbreviations**: CAPS - Clinician-Administered PTSD Scale; IES-R - Impact of Events Scale Revised; CBT - Cognitive Behavioral Therapy; MC - Minimal Contact; SCID - Structured Clinical Interview for the DSM-5; SIP - Structured Interview for PTSD; MVA - Motor Vehicle Accident; NR – Not Reported; PCL - PTSD Checklist; TMR – Traumatic Memory reactivation; RT - Reconsolidation Therapy; TAU - Treatment As Usual; PSS-I - PTSD Symptom Scale Interview; HTQ - Harvard Trauma Questionnaire; CET - Cognitive/Exposure Therapy; ER - Extended Release; DTS - Davidson Trauma Scale; PE - Prolonged Exposure; WTC - World Trade Center; VRE - Virtual Reality Exposure; PDS - PTSD Diagnostic Scale; CT - Cognitive Therapy; CR - Cognitive Restructuring; CPT - Cognitive Processing Therapy; CGI-S - Clinical Global impressions Severity scale; PCT - Present-Center Therapy; MDMA-AT - MDMA-Assisted Therapy; MP - Manualized Psychotherapy; CBCT - Cognitive Behavioral Conjoint Therapy; EMDR - Eye Movement Desensitization and Reprocessing
