# Supplementary material for: MDMA-assisted therapy and current treatment options for chronic, treatment-resistant, moderate or higher severity post-traumatic stress disorder: Systematic literature review
Source: PLoS One. 2025 Jul 16;20(7):e0327778. doi: 10.1371/journal.pone.0327778 (PMC12266454; doi:10.1371/journal.pone.0327778)
Supplement: S4.Table — (DOCX) [file pone.0327778.s004.docx]

**S4 Table**. **AEs captured in ≥2 studies of pharmacological interventions for PTSD, reported in ≥5.0% of treatment arms**

| **Adverse Event** | **FDA-Approved Medications** | | | | | | | | **Off-Label Medications** | | | | | |
| --- | --- | --- | --- | --- | --- | --- | --- | --- | --- | --- | --- | --- | --- | --- |
|  | **Paroxetine** | | | **Sertraline** | | | | | **Ketamine** | | **Divalproex** | **Nabilone** | **Ganaxolone** |  |
|  | Brunet, 2021  (N= 23)[93] | Seo, 2011 (N=20)[55] | Marshall, 2001 (N=365)[52] | Li, 2017 (N=36)[89] | Panahi, 2011 (N=35)[90] | Zohar, 2002 (N=23)[56] | Davidson, 2001 (N=98)[57] | Davidson, 2006 (N=173)[59] | Abdallah, 2022 (N= 53, 0.2 mg/kg)[79] | Abdallah, 2022 (N= 51, 0.5 mg/kg)[79] | Davis, 2008 (N=41)[80] | Jetly, 2008 (N=10)[81] | Rasmusson, 2017 (N=59)[82] | |
| Agitation | NR | 0.0% | NR | NR | NR | NR | NR | NR | 5.7% | 5.9% | NR | NR | NR | |
| Anxiety | NR | 15.0% | NR | NR | NR | NR | NR | NR | 3.8% | 3.9% | NR | NR | NR | |
| Asthenia | NR | NR | >10% | 19.4% | 16.0% | 17.0% | NR | NR | NR | NR | NR | NR | NR | |
| Constipation | NR | 5.0% | NR | 19.4% | 16.0% | NR | NR | 6.9% | 0.0% | 5.9% | NR | NR | NR | |
| Decreased appetite | NR | NR | NR | 16.7% | 16.0% | 13.0% | 12.0% | 7.5% | NR | NR | NR | NR | NR | |
| Decreased libido | NR | NR | >10% | NR | 13.0% | NR | NR | NR | NR | NR | NR | NR | NR | |
| Depression | NR | NR | NR | NR | NR | NR | NR | NR | 1.9% | 2.0% | NR | NR | NR | |
| Diarrhea | NR | NR | >10% | 13.9% | 22.0% | NR | 28.0% | 27.2% | 5.7% | 3.9% | 12.0% | NR | NR | |
| Dizziness | 8.7% | NR | NR | NR | NR | NR | NR | 12.1% | NR | NR | 24.0% | NR | NR | |
| Drowsiness | NR | NR | NR | 25.0% | 16.0% | 26.0% | 17.0% | NR | NR | NR | 12.0% | NR | NR | |
| Dry mouth | NR | NR | NR | 22.2% | 19.0% | 13.0% | 10.0% | 15.0% | NR | NR | NR | 40.0% | NR | |
| Fatigue | NR | 10.0% | NR | NR | NR | NR | 13.0% | 13.9% | 5.7% | 7.8% | NR | NR | NR | |
| Headache | 8.7% | NR | NR | 30.6% | 31.0% | 26.0% | 33.0% | 32.9% | 26.4% | 11.8% | 10.0% | 60.0% | 19.0% | |
| Increased appetite | NR | NR | NR | NR | NR | 13.0% | NR | NR | NR | NR | NR | NR | NR | |
| Increased urination | NR | NR | NR | NR | NR | NR | NR | NR | NR | NR | 10.0% | NR | NR | |
| Insomnia | NR | 10.0% | NR | 27.8% | 31.0% | NR | 35.0% | 10.4% | NR | NR | NR | NR | NR | |
| Irritability/Nervousness | NR | NR | NR | NR | NR | NR | 14.0% | NR | 9.4% | 5.9% | NR | NR | NR | |
| Nasal or sinus congestion | NR | NR | NR | NR | NR | NR | NR | NR | NR | NR | NR | NR | NR | |
| Nausea | NR | NR | >10% | 33.3% | 31.0% | 35.0% | 23.0% | 22.5% | 15.1% | 15.7% | 14.0% | NR | NR | |
| Paresthesia | NR | NR | NR | NR | NR | NR | NR | NR | NR | NR | NR | NR | NR | |
| Sedation | NR | NR | NR | NR | NR | NR | NR | NR | NR | NR | NR | NR | NR | |
| Somnolence | NR | NR | >10% | NR | NR | NR | NR | 10.4% | NR | NR | NR | NR | 7.1% | |
| Suicidality | NR | NR | NR | NR | NR | NR | NR | NR | 1.9% | 2.0% | NR | NR | NR | |
| Sweating | NR | NR | NR | NR | NR | NR | NR | NR | 1.9% | 3.9% | NR | NR | NR | |
| Tremor | NR | NR | NR | NR | NR | NR | NR | NR | NR | NR | NR | NR | NR | |
| Weight gain | NR | NR | NR | NR | NR | NR | NR | NR | NR | NR | NR | NR | NR | |

***Note:*** *Thirst, skin rash, palpitations, dysgeusia, and disturbance in attention were not reported in any of the trials in this part of Table S2*

**S4 Table**. **AEs captured in ≥2 studies of pharmacological interventions for PTSD, reported in ≥5.0% of treatment arms (cont.)**

| **Adverse Event** | **Off-Label Medications** | | | | | | | | | | | |
| --- | --- | --- | --- | --- | --- | --- | --- | --- | --- | --- | --- | --- |
|  | **Fluoxetine** | | | | **Prazosin** | | | **Cyclobenzaprine** | | **Mirtazapine** | | **Olanzapine** |
|  | Martenyi, 2006 (N=110)[70] | Martenyi, 2007 (N=163, 20 mg)[71] | Martenyi, 2007 (N=160, 40 mg)[71] | Barnett, 2002 (N=33)[96] | Raskind, 2007 (N=16)[62] | Raskind, 2013 (N=32)[63] | Raskind, 2018 (N=152)[60] | Sullivan, 2021 (N=90, low dose)[84] | Sullivan, 2021 (N=49, high dose)[84] | Davidson, 2003 (N=17)[91] | Seo, 2010 (N=20)[55] | Carey, 2012 (N=15)[83] |
| Agitation | NR | NR | NR | NR | NR | NR | NR | NR | NR | NR | 20.0% | NR |
| Anxiety | NR | NR | NR | NR | NR | NR | NR | NR | NR | NR | 15.0% | 6.7% |
| Asthenia | NR | NR | NR | NR | NR | NR | 36.2% | NR | NR | NR | NR | NR |
| Constipation | NR | NR | NR | NR | NR | NR | NR | NR | NR | NR | 0.0% | NR |
| Depression | NR | NR | NR | NR | 0.0% | 0.0% | 9.2% | NR | NR | NR | NR | NR |
| Diarrhea | 5.5% | NR | NR | 51.0% | NR | NR | 5.9% | NR | NR | NR | NR | 20.0% |
| Dizziness | NR | NR | NR | NR | 56.3% | NR | 48.0% | NR | NR | NR | NR | 13.3% |
| Drowsiness | NR | NR | NR | NR | NR | 3.1% | NR | NR | NR | NR | NR | NR |
| Dry mouth | 7.3% | NR | NR | NR | 12.5% | NR | 3.9% | 4.3% | 16.0% | NR | 5.0% | 13.3% |
| Fatigue | NR | NR | NR | NR | NR | NR | NR | NR | NR | NR | 15.0% | NR |
| Headache | 15.5% | 16.0% | 18.8% | NR | 18.8% | NR | 35.5% | 5.4% | 12.0% | NR | NR | 46.7% |
| Increased appetite | NR | NR | NR | NR | NR | NR | NR | NR | NR | 35.3% | NR | 40.0% |
| Increased urination | NR | NR | NR | NR | NR | NR | 21.7% | NR | NR | NR | NR | NR |
| Insomnia | 14.5% | NR | NR | NR | 6.3% | NR | 21.7% | 7.5% | 6.0% | NR | 0.0% | 20.0% |
| Irritability/Nervousness | 5.5% | NR | NR | NR | NR | NR | NR | NR | NR | NR | NR | NR |
| Nasal or sinus congestion | NR | NR | NR | NR | 37.5% | 21.9% | 37.5% | NR | NR | NR | NR | NR |
| Nausea | 12.7% | 12.9% | 13.8% | 57.0% | NR | NR | 30.9% | NR | NR | NR | NR | NR |
| Paresthesia | NR | NR | NR | NR | NR | NR | NR | 16.1% | 4.0% | NR | NR | NR |
| Sedation | NR | NR | NR | NR | NR | NR | NR | 2.2% | 12.0% | NR | NR | 73.3% |
| Somnolence | NR | 9.2% | 11.9% | NR | NR | NR | 33.6% | 11.8% | 16.0% | NR | NR | NR |
| Suicidality | NR | NR | NR | NR | NR | NR | 7.9% | NR | NR | NR | NR | NR |
| Sweating | NR | NR | NR | NR | 0.0% | NR | NR | NR | NR | NR | NR | 20.0% |
| Weight gain | NR | NR | NR | NR | NR | NR | NR | NR | NR | 17.6% | 5.0% | 93.3% |
| Palpitations | NR | NR | NR | NR | NR | 6.3% | 21.1% | NR | NR | 0.0% | NR | NR |
| Skin rash | NR | NR | NR | 6.0% | NR | NR | NR | NR | NR | NR | NR | 13.0% |
| Thirst | NR | NR | NR | 51.0% | NR | NR | NR | NR | NR | NR | NR | NR |

***Note:*** *Decreased libido, decreased appetite, tremor, dysgeusia, and disturbance in attention were not reported in any of the trials in this part of Table S2*

**S4 Table.** **AEs captured in ≥2 studies of pharmacological interventions for PTSD, reported in ≥5.0% of treatment arms (cont.)**

| **Adverse Event** | **Off-Label Medications** | | | | | | | | | | |
| --- | --- | --- | --- | --- | --- | --- | --- | --- | --- | --- | --- |
|  | **Risperidone** | **Tiagabine** | **Topiramate** | | **Venlafaxine** | | | **Eszopiclone** | | **Mifepristone** |  |
|  | Krystal, 2011 (N=133)[68] | Davidson, 2007 (N=114)[87] | Yeh, 2011 (N=17)[72] | Monga, 2023 (N=34)[73] | Davidson, 2006 (N=161)[74] | Davidson, 2006 (N=179)[59] | Pollack, 2011 (N=12)[77] | | Dowd, 2020 (N=13)[78] | Golier, 2023 (N=41)[86] |  |
| Anxiety | NR | NR | NR | NR | NR | NR | NR | | NR | NR |  |
| Asthenia | NR | NR | NR | NR | NR | NR | NR | | NR | NR |  |
| Constipation | NR | NR | NR | NR | 12.4% | 11.7% | NR | | NR | NR |  |
| Decreased appetite | NR | NR | NR | 14.7% | NR | 11.7% | NR | | NR | NR |  |
| Decreased libido | 6.1% | NR | NR | NR | 5.0% | NR | NR | | NR | NR |  |
| Depression | NR | NR | NR | NR | NR | NR | NR | | NR | NR |  |
| Diarrhea | NR | NR | NR | 20.6% | NR | 12.3% | NR | | NR | NR |  |
| Dizziness | NR | 32.0% | NR | NR | 18.0% | 13.4% | NR | | NR | 4.9% |  |
| Drowsiness | NR | NR | NR | NR | NR | NR | NR | | NR | NR |  |
| Dry mouth | NR | NR | NR | NR | 13.0% | 19.0% | NR | | NR | 4.9% |  |
| Fatigue | 13.7% | NR | NR | 20.6% | 8.1% | 10.6% | NR | | NR | 7.3% |  |
| Headache | NR | 25.0% | 11.0% | 23.5% | 28.6% | 29.6% | 12.0% | | 20.0% | 4.9% |  |
| Increased appetite | NR | NR | NR | NR | NR | NR | NR | | NR | NR |  |
| Increased urination | NR | NR | NR | NR | NR | NR | NR | | NR | NR |  |
| Insomnia | NR | NR | 23.0% | 20.6% | 7.5% | 13.4% | NR | | NR | NR |  |
| Irritability/Nervousness | NR | NR | 11.0% | NR | NR | NR | NR | | NR | NR |  |
| Nasal or sinus congestion/nasopharyngitis | 4.6% | NR | NR | NR | 5.0% | NR | NR | | NR | NR |  |
| Nausea | NR | 18.0% | NR | 17.6% | 21.7% | 25.1% | NR | | NR | NR |  |
| Paresthesia | NR | NR | 17.0% | 35.3% | NR | NR | NR | | NR | NR |  |
| Sedation | NR | NR | NR | NR | NR | NR | 16.0% | | 20.0% | NR |  |
| Somnolence | 9.9% | 20.0% | 23.0% | 17.6% | 5.6% | 11.7% | NR | | NR | NR |  |
| Suicidality | NR | NR | NR | NR | NR | NR | NR | | NR | NR |  |
| Sweating | NR | NR | NR | NR | 13.0% | NR | NR | | NR | NR |  |
| Tremor | NR | NR | NR | NR | 6.2% | NR | NR | | NR | NR |  |
| Weight gain | 15.3% | NR | NR | NR | 12.0% | NR | NR | | NR | NR |  |
| Dysgeusia | NR | NR | NR | NR | NR | NR | 32.0% | | 30.0% | NR |  |
| Disturbance in attention | 6.9% | NR | 11.0% | NR | NR | NR | NR | | NR | NR |  |
| Skin rash | NR | NR | NR | NR | NR | NR | NR | | NR | 4.9% |  |
| Thirst | NR | NR | NR | 14.7% | NR | NR | NR | | NR | NR |  |

**Note:** Agitation and palpitations were not reported in any of the trials in this part of Table S2
